# Carbon Thin‐Film Electrodes as High‐Performing Substrates for Correlative Single Entity Electrochemistry

**DOI:** 10.1002/smtd.202400639

**Published:** 2024-08-19

**Authors:** Marc Brunet Cabré, Christian Schröder, Filippo Pota, Maida A. Costa de Oliveira, Hugo Nolan, Lua Henderson, Laurence Brazel, Dahnan Spurling, Valeria Nicolosi, Pietro Martinuz, Mariangela Longhi, Faidra Amargianou, Peer Bärmann, Tristan Petit, Kim McKelvey, Paula E. Colavita

**Affiliations:** ^1^ School of Chemistry Trinity College Dublin Dublin 2 Ireland; ^2^ School of Chemistry, CRANN and AMBER Research Centres Trinity College Dublin Dublin 2 Ireland; ^3^ Dipartimento di Chimica Università degli Studi di Milano Via Golgi 19 Milano 20133 Italy; ^4^ Helmholtz‐Zentrum Berlin für Materialienund Energie GmbH (HZB) Albert‐Einstein‐Straße15 12489 Berlin Germany; ^5^ MacDiarmid Institute for Advanced Materials and Nanotechnology School of Chemical and Physical Sciences Victoria University of Wellington Wellington 6012 New Zealand

**Keywords:** 2D materials, carbon, correlative electrochemistry, mxenes, scanning electrochemical cell microscopy, single‐entity electrochemistry, X‐ray microscopy

## Abstract

Correlative methods to characterize single entities by electrochemistry and microscopy/spectroscopy are increasingly needed to elucidate structure‐function relationships of nanomaterials. However, the technical constraints often differ depending on the characterization techniques to be applied in combination. One of the cornerstones of correlative single‐entity electrochemistry (SEE) is the substrate, which needs to achieve a high conductivity, low roughness, and electrochemical inertness. This work shows that graphitized sputtered carbon thin films constitute excellent electrodes for SEE while enabling characterization with scanning probe, optical, electron, and X‐ray microscopies. Three different correlative SEE experiments using nanoparticles, nanocubes, and 2D Ti_3_C_2_T*_x_* MXene materials are reported to illustrate the potential of using carbon thin film substrates for SEE characterization. The advantages and unique capabilities of SEE correlative strategies are further demonstrated by showing that electrochemically oxidized Ti_3_C_2_T*_x_* MXene display changes in chemical bonding and electrolyte ion distribution.

## Graphical abstract

Graphitized carbon thin film electrodes are demonstrated as high‐performing substrates for correlative scanning electrochemical cell microscopy (SECCM). Three different types if nano‐entities are used to test performance, namely metal@C nanoparticles, carbon nanocubes, and 2D MXenes. Versatility is exemplified by correlative‐SECCM experiments that combine scanning probe, optical, electron, and X‐ray microscopies (SXM) in transmission and total electron yield modes.

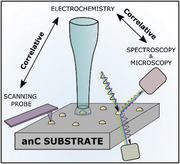

## 1 Introduction

Single‐entity electrochemistry (SEE) is an emerging area of research that aims at evaluating the electrochemical response of materials at the micro‐ and nanoscale.^[^ ^1^ ^]^ Several SEE studies have demonstrated how valuable this approach is toward achieving a fundamental understanding of the intrinsic electrochemical properties of nanomaterials, in particular with relevance to energy storage or energy conversion.^[^ ^2^ ^]^ Nanomaterials of interest for such applications often exhibit heterogeneity in their composition and structure that arises from population heterogeneity or from the presence of different nanoscale sub‐domains within single entities. This represents a significant challenge when interpreting their electrochemical response using conventional bulk electrochemistry, given that crucial information on, e.g., the role of specific catalytic sites or of inert sub‐domains can be obscured in the ensemble response of a macroscopic electrode.

**Figure 1:**
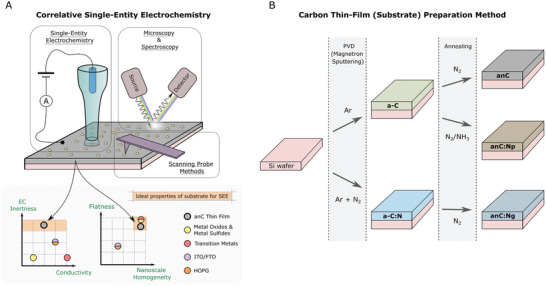
A) Top: Scheme of a correlative single entity electrochemistry (SEE) experiment; SEE demands that electrochemical, microscopic/spectroscopic and/or scanning probe characterization be carried out on the same sample and entity in a complementary manner. Bottom: Qualitative representation of desirable and undesirable properties of substrates for correlative SEE with relative positions of common SEE substrates. B) Schematic of the fabrication of anC, anC:N_P_, and anC:N_G_ substrates.

SEE in combination with complementary characterization techniques has opened the door to a new type of characterization known as correlative‐SEE^[^ ^3^ ^]^ that holds exceptional potential toward understanding nanomaterials for energy applications. In such methods, spectroscopy and/or microscopy are used in tandem with SEE to correlate the electrochemical response to chemical and/or structural properties of probed entities. Among the range of SEE methodologies currently available, scanning electrochemical cell microscopy (SECCM) stands out as particularly well‐suited for correlative‐SEE. First, SECCM shares similar sample preparation methods with typical characterization techniques, involving the immobilization of nano‐entities on a substrate surface, as illustrated in **Figure** **1**A. Therefore, it is possible to, in principle, seamlessly locate and characterize the probed entities with spectroscopic/microscopic methods to achieve correlation. Second, in SECCM a nanoscale meniscus establishes the electrical contact at discrete points to achieve electrochemical measurements with high spatial resolution. Considering that resolutions achievable can be as low as tens of nm,^[^ ^1d^ ^]^ SECCM can potentially deliver correlative‐SEE not only at the single entity level but at the sub‐domain level within a nanomaterial.^[^ ^2^, ^4^ ^]^ In such instances, localized wetting is highly advantageous as it preserves the majority of the nanomaterial in a pristine state, allowing for meaningful comparisons between probed and unprobed domains.

**Figure 2:**
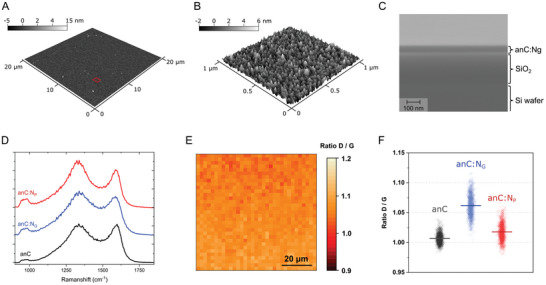
A) AFM image of anC:N_G_ substrate; red section indicates the approximate region that is expanded in B); both images show a smooth and homogeneous substrate surface. C) Cross section SEM image of the anC:N_G_ substrate: Si, SiO_2_ and anC:N_G_ layers can be observed in side view, with the anC:N_G_ layer displaying constant thickness and no evidence of inhomogeneities. D) Comparison of Raman spectra of anC, anC:N_G_ and anC:N_P_ displaying the characteristic D and G bands of amorphous carbons, as previously discussed.^[^ ^13b^ ^]^ E) Example of Raman mapping of the D/G intensity ratio across a typical anC:N_G_ surface and F) summary of D/G value distributions for all carbon materials.

Integration of SEE with other characterization techniques, however, remains challenging as correlative‐SEE measurements often necessitate to adapt samples and probes to ensure the fulfilment of various and potentially conflicting experimental requirements. Several studies have addressed and discussed the design of probe contact configurations of SECCM probes optimized for correlative measurements.^[^ ^2^, ^5^ ^]^ However, the design and optimization of novel substrates has received comparatively less attention despite these also playing a critical role in enabling nanomaterial dispersion/immobilization as well as multiple probing approaches. Some of the common requirements to achieve correlative‐SECCM include smooth topography to deliver good nanomaterial dispersions and controlled height‐contrast; as well as chemical homogeneity to deliver chemical contrast in spectroscopy. Good conductivity, electrochemical inertness and, preferably, low capacitive backgrounds are also paramount to facilitate SEE over a wide potential window. Carbon electrode materials generally fulfil these requirements, which are particularly important for electrocatalysis studies of nanomaterials. For instance, glassy carbon (GC),^[^ ^2^, ^5^, ^6^ ^]^ highly ordered pyrolytic graphite (HOPG)^[^ ^7^ ^]^ and boron‐doped diamond (BDD)^[^ ^8^ ^]^ have all been used as substrates for correlative SECCM with scanning electron microscopy (SEM); however, substrate thickness and opacity can limit applicability in correlative strategies that require probing through the substrate. Indeed, in the case of correlative‐SEE based on optical microscopy (OM) the substrate must also be transmissive/reflective in the energy range of relevance.^[^ ^3^, ^9^ ^]^ Indium tin oxide (ITO) thin films on glass are widely used for this purpose thanks to high conductivity and low absorptivity;^[^ ^3^, ^10^ ^]^ however, their electrochemical inertness is limited by metal leaching and redeposition processes,^[^ ^10^, ^11^ ^]^ thus restricting broad applicability in electrocatalysis. Finally, for batch correlative‐SEE measurements, e.g. correlative‐SEE across a sample library,^[^ ^12^ ^]^ multiple substrates of identical characteristics are often required. Then, the utilization of substrates that can be batch fabricated expands the possibilities for correlative‐SEE experimental designs.

**Figure 3:**
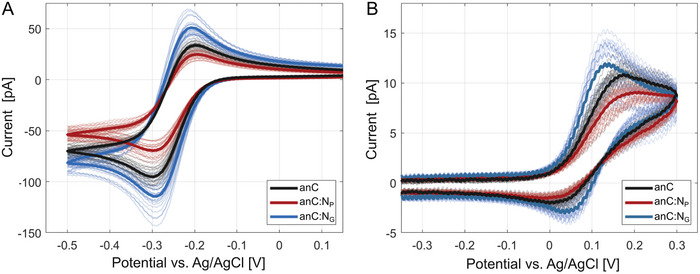
A) Voltammograms of outer‐sphere, [Ru(NH_3_)_6_]^3+/2+^, and B) [Fe(CN)_6_]^3‐/4−^ redox probes on bare anC, anC:N_G_, and anC:N_P_. Thin lines correspond to the CV for each of the 25 points probed in a grid; thick lines represent the mean response calculated for each substrate. All voltammograms were collected at 0.5 V s^−1^ with electrolyte of 4.0 mm Ru(NH_3_)_6_^3+^ in 20 mm KCl, and 1.0 mm Fe(CN)_6_^3‐^ in 10 mm KCl, for A and B respectively.

In this study, we discuss properties and demonstrate applications of graphitized carbon thin film electrodes as substrates for correlative‐SECCM. We first discuss chemical and structural properties of these films and how they can be tuned through synthesis/deposition conditions to deliver several of the above mentioned requirements of correlative‐SECCM. We demonstrate the capability and versatility of these substrates using three nano‐entities of very distinct morphological and chemical composition, such as carbon‐encapsulated nickel nanoparticles (Ni@C), carbon nanocubes (CNC), and 2D MXenes (Ti_3_C_2_T*_x_*). Correlative‐SEE of these was achieved by coupling SECCM with a range of widely accessible scanning microscopies, including scanning electron microscopy (SEM), energy‐dispersive spectroscopy (EDS), and atomic force microscopy (AFM). Finally, we demonstrate correlative‐SEE applications that integrate advanced synchrotron techniques such as scanning X‐ray microscopy (SXM) in transmission and total electron yield (TEY) modes. Using SXM we show that it is possible to obtain both nm‐resolution imaging and spectroscopic chemical information from X‐ray absorption spectra (XAS) on these thin films substrates to correlate against the electrochemical response of nano‐entities.

## 2 Results

### 2.1 Structural and Nanoelectrochemical Characterization of Carbon Thin Film Substrates

Correlative‐SEE benefits from access to substrates with a smooth and homogeneous morphology to enable resolution and characterization without hindrance/interference arising from substrate features. Three types of graphitized thin film carbon substrates were synthesized in this work as described in the experimental section and schematically illustrated in Figure 1B. The substrates were prepared via sputtering deposition followed by annealing under either inert or reactive gas flows to achieve graphitization^[^ ^13^ ^]^ yielding batches of three different carbon films: anC, a nitrogen‐free graphitized carbon; and anC:N_G_ and anC:N_P_, two different types of nitrogenated graphitized carbons. Morphological characterization of anC, anC:N_P_, and anC:N_G_ was carried out by AFM and SEM. **Figure** **2**A,B show representative AFM images over 400 and 1 µm^2^ areas, respectively, of an anC:N_G_ substrate; the morphology of these carbon surfaces is highly homogeneous, with slight local irregularities consistent with typical levels of particulate contamination resulting from fabrication outside of a cleanroom environment.^[^ ^14^ ^]^ The root mean square (RMS) roughness calculated over the 1 µm^2^ domains is 1.4 nm, while the thickness is <100 nm, as previously characterized.^[^ ^13a^ ^]^ Similar results are obtained on anC and anC:N_P_ substrates as shown in Figures S1 and S2 (Supporting Information) and summarized in **Table** **1**. Figure 2C shows the cross section of a sample obtained via SEM, where the Si, SiO_2_, and anC:N_G_ layers can be clearly distinguished. Figure S3 (Supporting Information) displays additional representative SEM cross sections for anC, anC:N_G_, and anC:N_P_ substrates at different magnifications. As in the case of AFM images, SEM demonstrates excellent uniformity across annealed carbon layers, with an overall featureless surface and an absence of local defects/inhomogeneities, thus satisfying requirements for their application in correlative SEE.

**Table 1:** Roughness (RMS) and thickness of carbon thin film substrates tested for SEE. Composition (atomic‐%), proportion of graphitic (N_G_), pyridinic (N_P_) and pyrrolic (N_Pyrr_) functionalities contributing to N 1s spectra, and %‐content of trigonally bonded carbons ($C_{sp^{2}}/C_{\mathrm{tot}}$) were obtained from XPS spectra, as discussed in references.^[^ ^13^ ^]^

Carbon Thin film	RMS (nm)	*D* (nm)	Components [at.%]			C 1s [%]	N 1s [at.%]		
			C	N	O	$C_{sp^{2}}/C_{\mathrm{tot}}$	N_G_	N_Pyrr_	N_P_
anC	0.7	83 ± 1	96	–	4.0	68	–	–	–
anC:N_G_	1.4	65 ± 1	91	3.0	6.0	68	60	6.0	34
anC:N_P_	1.4	65 ± 1	94	0.8	5.2	71	16	31	53

The chemical composition of these materials has been investigated and results have been reported in previous work from our group.^[^ ^13^ ^]^ Surface atomic composition and degree of graphitization were assessed via XPS and the results are summarized in Table 1. Briefly, all three films display similar degrees of graphitization as evidenced by the %‐contribution of trigonally bonded carbon to the overall C 1s high‐resolution spectra ($C_{sp^{2}}/C_{\mathrm{tot}}$). The nitrogen‐free carbon substrate displays a small O‐content, likely the result of atmospheric adsorbates (e.g., water) typically observed at carbon thin film surfaces after air exposure.^[^ ^15^ ^]^ The nitrogenated anC:N substrates display surface N‐functionalities which are predominantly pyrrolic/pyridinic‐N in the case of anC:N_P_, and predominantly graphitic‐N in the case of anC:N_G_ (Table 1). The compositional homogeneity of the carbon was supported by Raman spectroscopy and microscopy results. Figure 2D shows the Raman spectra of the three films deposited on a Si wafer showing the presence of a D band (ca. 1360 cm^−1^) and a G band (ca. 1590 cm^−1^) characteristic of amorphous and graphitized carbons; the peak at ca. 1000 cm^−1^ arises from the Si substrate. The D/G intensity ratio is diagnostic of carbon graphitization but also of the type of nitrogenated structures present at the surface. Figure 2E shows a Raman map of the D/G intensity ratio across the surface of anC:N_G_ that supports a high degree of uniformity, with D/G values narrowly distributed around the mean (Figure 2F; Figure S4, Supporting Information). This indicates that the three carbon films are structurally homogeneous across relatively large areas.

A homogeneous electrochemical response across the substrate surface is paramount to providing reliable electrochemical contrast in SEE methods. anC substrates were characterized electrochemically using SECCM by probing multiple domains of micron scale size (1–2 µm^2^). Electrochemical mappings were obtained using a standard redox probe that is outer‐sphere, such as Ru(NH_3_)_6_^3+/2+^, and a redox couple that can display sensitivity to surface chemistry at carbon electrodes, such as Fe(CN)_6_^3‐/4−^.^[^ ^16^ ^]^ A total of 25 independent points were probed across a 1600 µm^2^ region of the substrate, by utilizing a defined 5 × 5 point grid with 20 µm spacing between points. For each redox probe a single pipette probe was used to measure all 3 substrates consecutively, thus ensuring similar mass transport conditions at all probed points.^[^ ^12^ ^]^ **Figure** **3**A,B displays the cyclic voltammogram (CV) datasets obtained at 0.5 V s^−1^ on anC, anC:N_P_ and anC:N_G_ thin film substrates using 4.0 mm Ru(NH_3_)_6_^3+/2+^ and 1.0 mm Fe(CN)_6_^3‐/4−^. Figure 3A,B also display a comparison of the mean response for each of the substrates probed (thick traces), obtained by averaging across all 25 points in each grid. Individual plots for each dataset obtained on the three carbon substrates with the two types of redox probe are reported in Figures S5 and S6 (Supporting Information). The CVs show good reproducibility and narrowly dispersed currents; the small dispersion observed between points within the same substrate type for either of the redox probes is consistent with small variances to be expected in the size of the SECCM droplet established at each contact point. Figure S6 (Supporting Information) shows histograms of the limiting currents obtained at the three carbon substrates for both probes; these suggest a narrow spread in values with the mean limiting current smaller for anC:N_P_ compared to anC or anC:N_G_. These results indicate that anC, anC:N_G_ and anC:N_P_ can effectively provide a consistent and homogeneous electrochemical response across their surface for surface‐sensitive and outer‐sphere redox probes, making them suitable candidates for undertaking SEE measurements, as indicated by our previous work.^[^ ^2f^ ^]^

**Figure 4:**
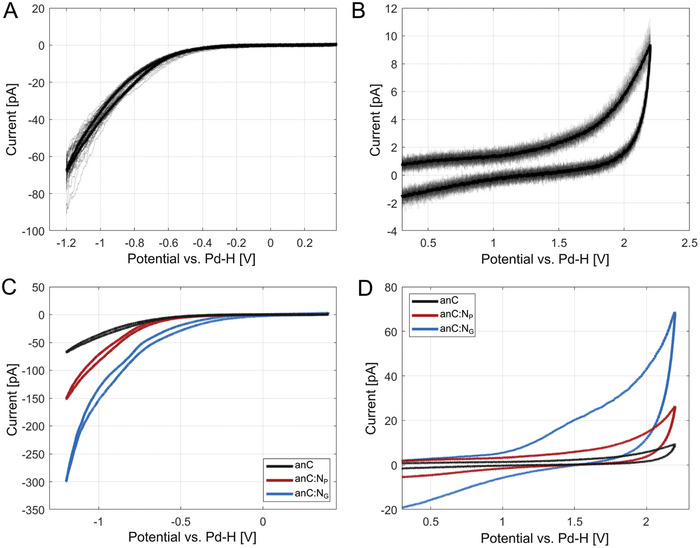
CVs obtained via SECCM in 20 mm H_2_SO_4_ supporting electrolyte at 0.5 V s^−1^ at the A) cathodic and B) anodic limits of the potential window using bare anC substrates as working electrodes. Thin lines correspond to CVs at each of the 25 grid points probed; thick lines represent the calculated mean response for the substrate. C,D) show a comparison of the mean voltammograms obtained using anC, anC:N_P_, and anC:N_G_ substrates at the cathodic and anodic limits, respectively, of the potential stability window.

The electrochemical response of the carbon substrates in supporting electrolyte is also of critical importance to provide good contrast in SEE methods. **Figure** **4**A,B shows CVs obtained at the anC substrate in 20 mm H_2_SO_4_ at 0.5 V s^−1^, at the cathodic and the anodic ends of the potential window, respectively; results for anC:N_G_ and anC:N_P_ are shown in Figure S7 (Supporting Information). Measurements at anodic and cathodic ends were carried out independently, thus ensuring that any substrate oxidation that might possibly take place at high potentials (> +2 V vs. SHE) does not affect the observed cathodic behavior. The narrow dispersion on the anodic and cathodic currents in Figure 4A,B indicates excellent homogeneity in the electrochemical responses of the carbon thin film substrates. For the anC substrate, CVs show a reproducibly wide potential window, from −0.5 to +1.95 V before the onset of any significant faradaic currents (threshold set at ± 5 pA). The cathodic limit is set by the onset of the hydrogen evolution reaction (HER), while the anodic limit is set by the oxygen evolution and/or carbon oxidation anodic currents.^[^ ^17^ ^]^ As showed in our prior work^[^ ^2f^ ^]^ and also later in the manuscript, the low and consistent HER activity observed on the anC substrate does not represent an impediment to providing clear contrast in SEE measurements at the cathodic end beyond overpotentials of ‐0.5 V vs. SHE.

Figure 4C,D show a comparison of the mean electrochemical response for the three substrates anC, anC:N_P_ and anC:N_G_. The presence of nitrogen functionalities in anC:N_G_ and anC:N_P_ results in enhanced HER currents relative to anC, as shown in our prior work on the characterization of these materials using macroscopic thin film electrodes.^[^ ^13b^ ^]^ At the anodic limit, larger faradaic currents as well as a larger capacitive response are observed for the two substrates containing nitrogen functionalities, relative to the N‐free anC substrate. This suggests that for SEE electrocatalysis studies, the anC substrate might offer the best performance in terms of electrochemical inertness and low capacitive background.

**Figure 5:**
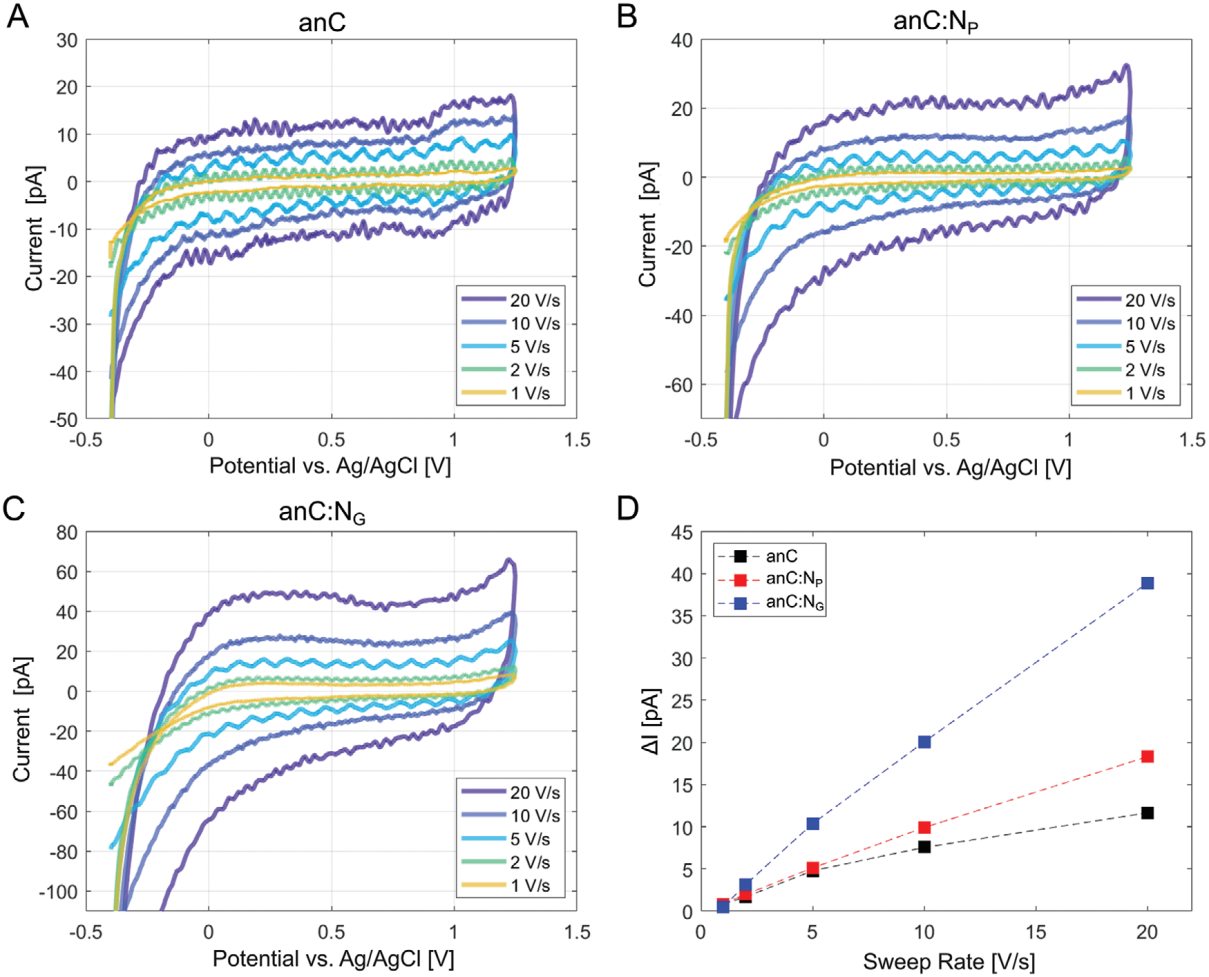
Mean CVs obtained at varying scan rate in 20 mm Na_2_SO_4_ at A) anC, B) anC:N_P_, and C) anC:N_G_ substrates; mean currents were calculated over 5 independently probed points in each SECCM grid. D) Plots of average capacitive currents versus scan rate for all three carbon thin film substrates. Average values were calculated from integrated forward and backward scans in the range 0 to 1.0 V; dashed lines have been included to guide the eye.

The capacitive behavior of the substrates was examined by performing CVs at varying scan‐rate (20, 10, 5, 2, and 1 V s^−1^) in 20 mm Na_2_SO_4_. **Figure** **5**A–C display the mean response obtained at each scan rate, calculated over 5 independently probed points. The CVs display the typical waveform of double layer charging; Figure 5D shows that capacitive currents display a linear response as a function of scan rate, while the mean capacitance appears to increase in the order anC < anC:N_P_ < anC:N_G_, likely as a result of a combination of differences in electronic structure, wettability and surface chemistry properties across the three substrates.

In summary, electrochemical characterization using SECCM methods over independent microscale domains at anC, anC:N_P_ and anC:N_G_ substrates indicates that these carbon thin films yield narrowly dispersed and homogenous current response. They were all found to provide sufficient conductivity to avoid distortions arising from resistance or impedance when probing at current magnitudes typical of SEE (pA to 10′s of nA). The anC substrate displays the widest potential window and smallest capacitive background across all three materials tested; therefore, the electrochemical performance of anC, together with its smooth topography (< 1 nm RMS roughness) and homogeneous chemical composition suggest that anC films have outstanding performance as SEE substrates.

### 2.2 Correlative SECCM at anC Substrates

To demonstrate the potential of anC substrates for correlative SEE via SECCM methods, three examples of correlative SEE are discussed in the following sections. All of these used anC as a substrate working electrode, while hyphenating SECCM studies with different microscopic/spectroscopic methods toward the characterization of three different types of nano‐entities: encapsulated Ni nanoparticles (Ni@C), carbon nanocubes and 2D Ti_3_C_2_T_x_ MXenes.

#### 2.2.1 Correlative SECCM of Ni@C Nanoparticles

Carbon encapsulation of transition metal nanoparticles has been identified as a promising strategy for the development of sustainable electrocatalysts.^[^ ^18^ ^]^ The carbon shell has been proposed to modulate adsorption of redox species, to improve tolerance to poisoning, and to reduce metal corrosion and/or aggregation. However, M@C composites are usually fabricated/synthesized using methods that result in heterogeneous populations of such nanostructures, leading to significant challenges in the interpretation of structure‐activity relations. Relating the ensemble electrochemical response to specific morphologies or carbon shell compositions is a complex task, because sub‐populations of nanomaterials, highly active sites and/or mass‐transport effects at nanostructured ensembles can often confound interpretation, as demonstrated for other nanomaterials.^[^ ^2^, ^4^, ^19^ ^]^

**Figure 6:**
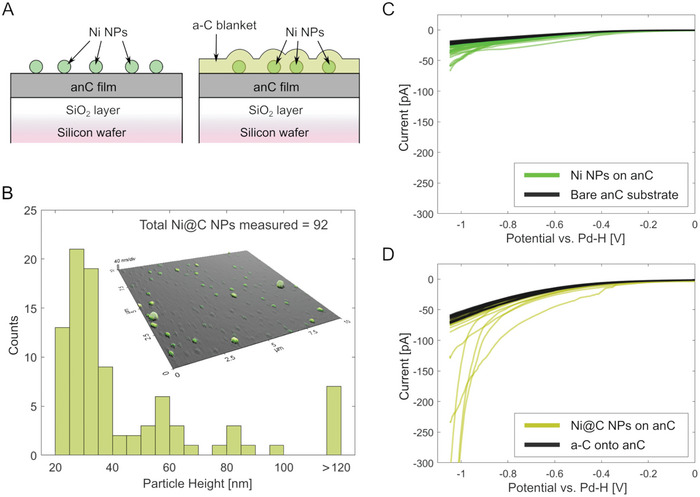
A) Scheme showing the architecture of samples measured via SECCM. Left: bare Ni NPs deposited over an anC substrate by drop‐casting. Right: Ni NPs deposited over anC and further encapsulated by an amorphous carbon (a‐C) shell yielding Ni@C structures. B) Distribution of Ni NP heights observed after drop‐casting over anC substrates via AFM; the inset shows an AFM image of the Ni@C nanostructures, evidencing that encapsulated Ni NPs can be clearly identified over the flat anC film. C) Voltammograms obtained in 0.1 m H_2_SO_4_ at each of the 36 points probed via SECCM for the sample prepared with bare Ni NPs on anC; the response of the bare anC substrate is shown in black for comparison. D) Voltammograms obtained at each of the 36 points probed via SECCM for the sample displaying Ni@C nanostructures; the response of the anC substrate coated with the same a‐C shell as the Ni NPs is shown in black for comparison.

Progress has been made in SEE methods for studying intrinsic activity at metal and metal oxide nanoparticles (NPs);^[^ ^19^, ^20^ ^]^ however, there are no reports to the best of our knowledge that achieve correlative SEE and physical characterization on M@C.^[^ ^3c^ ^]^ Here with the use of the anC carbon substrate, the effects of carbon encapsulation on the HER catalysis at Ni NP electrode materials were investigated by performing correlative characterization. Samples were prepared by first drop‐casting a Ni NP dispersion on the anC substrates; a carbon shell consisting of amorphous carbon (a‐C) was then deposited via magnetron sputtering,^[^ ^16a^ ^]^ yielding Ni@C nanostructures as shown in **Figure** **6**A.

AFM was used to image the Ni@C NPs obtained after coating the Ni NPs with a 10 nm thick a‐C layer and to obtain a size distribution of for deposited nanoparticles, as shown in AFM image of Figure 6B. (see also Figure S8, Supporting Information). The size distribution for Ni@C nanostructures (*N *= 92) obtained from multiple AFM images over a total sampled area of 400 µm^2^ (see Sections S1–S3, Supporting Information), shows that the majority of nanostructures have a height in the range 20–70 nm. This is consistent with the presence of discrete, isolated Ni@C nanoparticles over the substrate surface, given a nominal Ni NP average size of 50 nm provided by the manufacturer. Using image analysis over the same regions (see Section S3, Supporting Information for details), the probability of sampling an individual Ni@C nanostructure upon contacting these surfaces using a 1 µm^2^ probe was estimated to range between 14% and 28%. This result indicates that the Ni@C particle density across the substrate is sufficiently low to ensure that single entities can be probed electrochemically. Nonetheless, the probability estimates span a relatively large range, thus suggesting that drop‐casting yields inhomogeneous particle densities, so that the probability of sampling single particles upon contact might depend on the specific probed region of the surface.

To investigate the electrochemical performance of bare Ni and Ni@C NPs in the HER region, voltammograms were performed with an SECCM probe containing 0.1 m H_2_SO_4_. A single voltammogram cycle was recorded at each point of a 6 × 6 SECCM grid with 5 µm spacing between grid points. Further details of the SECCM measurements are provided in Section S4 (Supporting Information). All 36 cathodic sweeps recorded for a sample with encapsulated Ni particles (Ni@C), and for a sample without encapsulation of the Ni particles (bare Ni NPs) are reported in Figure 6C,D. The responses for the bare anC substrate and for the anC substrate with a 10 nm a‐C layer are also displayed in Figure 6C,D with black traces; these were collected in regions that were not modified via drop casting and were used for comparing the responses of regions with Ni NP. The presence of bare Ni NPs (Figure 6C) results only in small changes in the cathodic currents relative to the response of the bare substrate anC. Bare Ni NPs dissolve in sulfuric acid solutions^[^ ^21^ ^]^ so that surface activity is decreased due to metal leaching, translating into only modest enhancements in HER currents relative to the carbon substrate. On the other hand, several voltammograms recorded on Ni@C samples (Figure 6D) present significantly enhanced HER response. Although it is not possible to completely exclude Ni NP reconstruction after carbon coating, such changes are unlikely given that mild deposition protocols were used (no bias, high deposition gas pressure) and Ni carbides display poor stability.^[^ ^22^ ^]^ Therefore, these results strongly suggest that carbon shell encapsulation provides protection against Ni dissolution, while still preserving the advantages of a metal surface for the HER activity.

**Figure 7:**
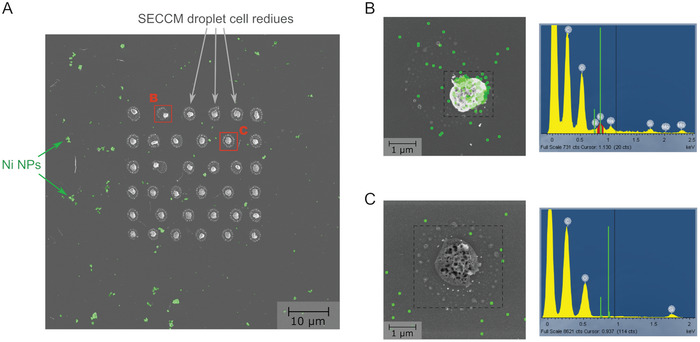
A) SEM image obtained after SECCM measurements on a Ni@C sample; droplet residues resulting from SECCM contacts are clearly seen to form a 6 × 6 grid pattern. Distinct features assigned to Ni NPs are shown in green. B) EDS map (green) overlaped over a high magnification SEM image of point B (i.e., point contacted in (A) that presents enhanced HER) showing the presence of Ni. C) EDS map (green) overlaped over a high magnification SEM image of point C (i.e., point contacted in (A) that presents HER response comparable to that of the anC substrate); the EDS map does not display Ni peaks or features characteristic of Ni NPs.

**Figure** **7**A shows an SEM image of the Ni@C sample after probing the sample via SECCM; the image shows Ni@C entities across the anC surface (see also Figure S12, Supporting Information) and the electrolyte residues in a 6 × 6 grid pattern resulting from contact with the nanopipette droplet. The mean droplet cell residue size of 8.5 ±  0.9 µm^2^ calculated from the images can be assumed to be equal to the size of the droplet cell.^[^ ^2f^ ^]^ The residues clearly identify the domains of the sample from which the electrochemical response originates, thus enabling further characterization and correlation with composition/morphology. As an example, two SECCM grid points, marked with frames B and C, were further characterized via EDS; point B corresponds to a point with enhanced HER, whereas C corresponds to a point that did not display HER current enhancements in Figure 6D (see also Figure S11, Supporting Information). Figure 7B,C shows high magnification SEM images of B and C, respectively, overlapped with the EDS mapping of the Ni peak; the corresponding integrated EDS spectra over the entire image are shown to the right of the two images. It is clear that the point that displays HER enhancements (B) also shows a high Ni content within the probed area, while the Ni peaks are negligible in the case of the point with no HER enhancement (C).

Thanks to the anC substrate that provided good contrast between single entity and substrate over SECCM, AFM, SEM, and EDS methods, correlative single entity electrochemical and physical characterization were achieved for carbon encapsulated Ni NPs. Probed points that displayed HER enhancements correlated positively with the presence of Ni@C nanostructures. Further support for this conclusion is provided by a comparison between the frequency of HER enhancement and particle statistics obtained from an analysis of the SEM images of the region surrounding the SECCM grid (see Section S4, Supporting Information). Given a probe contact area of 8.5 µm^2^, the probability of sampling a Ni@C nanostructure was estimated at 17 ± 3% over the regions immediately adjacent to the grid (see Figure S14 and Table S2, Supporting Information). This is in excellent agreement with 6 points out of 36 points yielding enhanced HER currents, i.e., 16.6% of the voltammetry curves. Our observations and analysis therefore strongly suggest that the a‐C coating/shell protects the Ni NP core from dissolution/leaching so that Ni@C nanostructures yield enhanced HER responses. We also observed that Ni@C nanostructures showed varying extents of enhancements relative to bare Ni NP; however, the origin of such variance cannot be identified on the basis of the above SECCM‐EDS experiments alone. Notably, very recent SECCM studies^[^ ^7a^ ^]^ of electrocatalysis at size‐controlled nanoparticles and aggregates also report significant variances in faradaic responses, while highlighting the challenges in discriminating the role played by chemical heterogeneity, NP‐support interactions and NP evolution within the droplet cell in determining the observed response. Our results using Ni@C nanostructures demonstrate the advantages of the approach presented in this work for investigating the contribution of M@C nanoparticle sub‐populations to activity enhancements, which would be extremely challenging to identify using macroelectrode techniques.

#### 2.2.2 Characterization of Carbon Nanocubes

**Figure 8:**
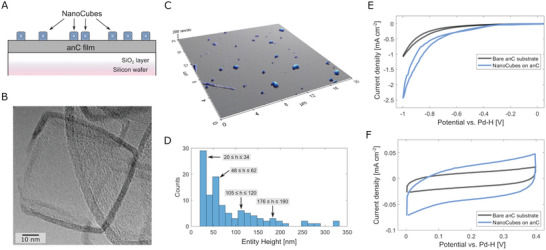
A) Scheme of the CNC/anC sample architecture, showing nanocubes drop‐cast over the anC substrate. B) TEM image of carbon nanocubes on anC showing polyhedral nanostructure and carbon walls consisting of multilayer graphene (ca. 11 layers). C) AFM image of nanocubes on an anC substrate, showing that individual CNC entities can be identified onto the smooth anC substrate; and D) height distribution obtained from the AFM image (*N* = 108). E) Voltammograms in 0.100 m H_2_SO_4_ at 0.020 V s^−1^ showing that nanocubes yield enhanced HER current densities compared to the bare anC substrate. F) Voltammograms in 0.100 m H_2_SO_4_ at 1.000 V s^−1^ in the double layer region of the potential window for anC and CNC/anC regions; both voltammograms display square‐like capacitive responses however the current densities are consistent with higher ECSA at CNC/anC samples.

Carbon nanomaterials of different dimensionality have been widely studied as materials for electrochemical applications, including via SEE approaches. Here an example of “carbon‐on‐carbon” single‐entity measurements is presented utilising carbon nanocubes (CNC) on annealed carbon substrates (anC). CNCs were deposited on anC substrates by drop‐casting resulting in anC‐supported nanostructures (CNC/anC); **Figure** **8**A shows a scheme of the CNC/anC sample architecture. Figure 8B shows a transmission electron microscopy (TEM) image of the CNCs used in our experiments. The image shows the characteristic polyhedral structure with clear edges and faces; based on thickness determinations, the faces consist of ≈11 graphene layers and the N‐content was in the range of 6–7%. From TEM determinations, the average size of each nanocube is estimated to be ≈50 nm. Figure 8C shows an AFM height‐image of a CNC/anC surface, displaying the presence of nanostructures dispersed on the anC substrate with the height distribution shown in Figure 8D. The majority of the nanostructures possess height <110 nm thus suggesting the presence of either isolated CNC or of CNC aggregates with height equivalent to that of 2–3 CNC entities.

Nano‐electrochemical characterization was performed by SECCM, achieving measurements on microscale domains (ca. 1 µm^2^) over sample areas with and without CNC deposited. The electrocatalytic response was evaluated via cyclic voltammetry at 0.020 V s^−1^ in 0.1 m H_2_SO_4_ over the −1.0 – 0 V potential region (vs Pd‐H reference electrode). Figure 8E shows enhanced HER current densities for a sample region with drop‐cast CNCs, compared to the response on the bare anC substrate. The capacitive response was evaluated via cyclic voltammetry scans at 1 V s^−1^ in 0.1 m KCl in the 0 – 0.4 V potential range, as shown in Figure 8F. Typical double layer capacitive currents are observed for both anC and CNC/anC surfaces; however, the current density obtained for CNC/anC is greater than that of bare anC regions, which is consistent with the CNCs presenting a larger electrochemically active specific surface area (ECSA). This experiment demonstrates that due to the homogeneity and topographic smoothness of annealed carbon thin film substrates it is possible to obtain electrochemical contrast in both faradaic and non‐faradaic potential windows using nano‐electrochemical methods, even in the challenging case of a “carbon‐on‐carbon” sample architecture.

#### 2.2.3 Correlative SXM and SECCM on 2D Ti_3_C_2_T_x_ MXenes

MXenes are 2D materials from the family of transition metal (M = Ti, V, W, etc.) carbides, nitrides, or carbonitrides. Their unique surface chemistry, given by terminal groups (T*_x_*), and their high conductivity make these materials outstanding candidates for several electrochemical applications, including energy storage, energy conversion and sensing.^[^ ^23^ ^]^ MXene properties depend on the flake size, shape and number of layers; as such, characterization at the few/single‐entity level is paramount for understanding their properties and optimising performance.^[^ ^3^, ^24^ ^]^ Nano‐impact^[^ ^24b^ ^]^ and SECCM^[^ ^2f^ ^]^ have been used to study individual MXene flakes however, as mentioned in the introduction, SECCM enables correlative characterization. In our prior study of Ti_3_C_2_T*_x_* MXenes, correlative nano‐electrochemical (SECCM) and morphological (SEM, AFM) characterization on single flakes was achieved.^[^ ^2f^ ^]^ However, to the best of our knowledge there are no reports of correlative electrochemical‐spectroscopic characterization at the single‐entity level on MXenes. X‐ray microscopies can provide spectroscopic information with sub‐micron resolution and our recent work demonstrated chemical mapping of MXenes with nm‐resolution via X‐ray photoelectron microscopy (X‐PEEM)^[^ ^25^ ^]^ and SXM in both transmission and TEY modes.^[^ ^26^ ^]^ Herein, we show that graphitized carbon thin films can satisfy stringent requirements of both SECCM and SXM^[^ ^27^ ^]^ to enable correlative mapping of MXene composition and electrochemical response.

**Figure 9:**
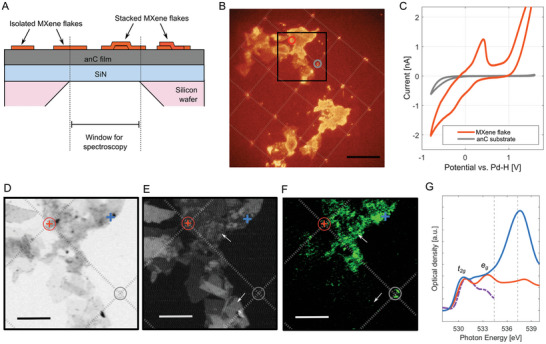
A) Scheme of the sample geometry that enables correlative STXM and SECCM characterization. The anC substrate was manufactured on a SiN window and the MXene flakes were deposited onto the substrate by drop‐casting. B) Optical image of a region probed via SECCM showing the grid points (dashed lines between points have been added as a visual guide) and the MXene flakes on top of the anC/SiN substrate. Scalebar: 15 µm C) Representative voltammograms acquired at points that contact the anC substrate (gray) and the MXene flake (orange), corresponding to positions circled in the same color in panel (B). D) Transmission and E) TEY SXM images of Ti_3_C_2_T*_x_* MXene at Ti L‐edge (463 eV) obtained over the region framed in black in the optical image. F) Transmission image acquired at 537.4 eV divided by the image at 534.4 eV to reveal the areas with sulfate ions. Arrow in (E) and (F) indicate region with opposite contrast change in TEY (E) and H_2_SO_4_ residue contrast (F). G) Transmission XA‐spectrum at O K‐edge for Ti_3_C_2_T*_x_* MXene, anodically oxidized in H_2_SO_4_ (orange), pristine (violet) and containing H_2_SO_4_ (blue) obtained at positions indicated with a cross in Figure Fand outside the grid for pristine MXene. Scalebar: 5 µm.

anC thin film substrates were fabricated on SiN membranes following the same protocols applied to standard Si wafer substrates. MXene (Ti_3_C_2_T*_x_*) dispersions were drop‐cast on the substrate, resulting in sample structures schematically shown in **Figure** **9**A. AFM images of single MXene flakes on smooth anC are shown in Figure S15 (Supporting Information). The electrochemical response of MXene flakes was evaluated by performing a 5 × 5 SECCM grid, with a spacing of 15 µm between points; 2 consecutive voltammograms were collected at each point using 10 mm H_2_SO_4_ as electrolyte. Microscopy imaging via optical, X‐ray transmission and TEY modes, was performed afterward over the regions scanned by SECCM. The probed points in the grid can be clearly identified, as in the optical image in Figure 9B, thanks to the electrolyte residue left by the nanopipette probe (see also Figure S16, Supporting Information), thus enabling correlation of the electrochemical response with the probed morphology.

Representative voltammograms in 10 mm H_2_SO_4_ electrolyte obtained at points that contact a MXene nanostructure (flake or a stack of flakes) and that contact the bare anC substrate are shown in Figure 9C. These voltammograms were obtained at points corresponding to the circles shown in Figure 9B; the potential sweep started at anodic potentials greater than +1 V vs. Pd‐H (i.e., > +0.95 V vs. SHE) and a prominent irreversible oxidation peak is observed in the case of the point that contacts the MXene flake. For potentials lower than +1 V vs. Pd‐H the cyclic voltammogram shape can be attributed to the characteristic MXene pseudocapacitive response in acid electrolyte.^[^ ^2^, ^28^ ^]^ In contrast, the voltammogram obtained on bare anC is mostly featureless across the entire potential window except for a small cathodic current below −0.5 V vs. Pd‐H, which can be attributed to the HER onset. A complete electrochemical dataset for all points probed in the SECCM grid, including the two consecutive voltammogram cycles measured at each position, is reported in Figure S17 (Supporting Information). The optical image of the SECCM grid displayed in Figure S16 (Supporting Information) can be used to correlate sample morphology to electrochemical response: notably, all MXene contacted points yield enhanced anodic currents relative to the bare anC substrate, albeit of varying magnitudes.

SXM was carried out over the region framed by a square in Figure 9B; additional images over other sample regions are displayed in Figures S18 and S19 (Supporting Information). Due to the conductivity and transmittance of the substrate, both transmission (Figure 9D) and TEY (Figure 9E) could be performed at the Ti L‐edge.^[^ ^26^ ^]^ The contrast in the transmission image results from the optical density (OD) depending on the X‐ray absorption cross section of the probed elements and the thickness of the sample, providing bulk‐sensitive imaging. In Figure 9D, MXene flakes of different thicknesses are clearly observed. Black nanoparticles of a few hundreds of nanometres are also observed over the MXenes, which could be related to small TiO_2_ nanoparticles formed upon local oxidation resulting from prolonged air and aqueous electrolyte exposure during the SECCM experiments. On the contrary, the TEY contrast results from the electrons emitted from the top MXene layer, depending on the surface work function and independent of thickness, providing surface‐sensitive imaging. While uniform electron emission is observed over the full flakes which were not contacted by the pipette (bottom region in Figure 9E indicated by arrow), as observed previously in pristine Ti_3_C_2_T*_x_* MXene,^[^ ^26^ ^]^ the TEY signal is much less uniform over the flake in contact with the pipette (top region in Figure 9E indicated by arrow). In our opinion, this change in the surface electron emission is due to the presence of a thin layer of residues from the H_2_SO_4_ electrolyte spreading over the flake, beyond the size of the droplet from the nanopipette.

This assumption is further confirmed by measuring the transmission SXM at the oxygen K‐edge (Figure 9G). No TEY could be recorded at the O K‐edge, probably due to the lower electron emission at the O K‐edge compared to the Ti L‐edge. The XA spectra at the O K‐edge obtained at the position probed by the SECCM (orange spectrum at orange cross position in Figure 9F) and a neighbouring position on the same flake (blue spectrum at blue cross position in Figure 9F) are shown in Figure 9G. A peak is clearly observed at 537.6 eV associated with the absorption band of the O 2p antibonding molecular orbitals of sulfate ions,^[^ ^29^ ^]^ confirming the presence of anions from the electrolyte at both positions. Interestingly, the sulfate band was found to have higher absorption at the position not in contact with the SECCM probe (blue cross). The distribution of the anions can be mapped by dividing the SXM image recorded at the maximum of the sulfate absorption (537.6 eV) to the absorption below this band (534.4 eV), as shown in Figure 9F in green contrast. Sulfate residues from the SECCM droplet on the anC film (gray circle) are clearly identified in Figure 9F. On the other hand, the droplet in contact with the MXene (orange circle) is not clearly visible. Instead, significant X‐ray absorption (green contrast) is observed all over the MXene flakes up to ∼10 microns away from the droplet. This may result from the hydrophilic nature of the MXene surface, facilitating the spreading of the electrolyte, or the electrophoretic displacement of ions upon the application of an anodic potential. We speculate that the ion distribution and localization might be affected by cycling conditions and/or potential limits used for SECCM probing. Note that no absorption is observed on MXene flakes which were far away from the droplet or spatially isolated from the contacted flake. A good correlation between the TEY at the Ti L‐edge and the difference image at the O K‐edge is observed. The high surface sensitivity of the TEY at the Ti L‐edge associated with the high chemical sensitivity at the O K‐edge suggest that sulfate ions are found on the top of the MXene layer contacted with the SECCM droplet.

In addition, the local oxidation of the MXene induced by the anodic potential applied by the nanopipette can also be monitored by XAS at the O K‐edge (Figure 9G). An enhancement of the peak at 533.6 eV, attributed to Ti‐O orbital mixing associated with *e_g_* symmetry (orange spectrum), is observed when compared to a pristine MXene flake not in contact with the nanopipette (violet spectrum). This suggests greater local oxidation of the MXene flake at electrochemically probed positions,^[^ ^26^ ^]^ in good agreement with the SECCM voltammograms showing large anodic currents at potentials greater than +1.0 V vs. Pd‐H (Figure 9C).

Further experiments as a function of scan rate, potential window and electrolyte composition are needed to understand local electrochemical oxidation processes and ion diffusion on MXene flakes; however, it is clear that correlative SXM‐SECCM can reveal unique insights on ion transport that are challenging to discern using the two methods in isolation. Combined SXM‐SECCM using substrates tailored for both conductivity and high transmittance with soft X‐rays offers a powerful route to revealing local and non‐local effects arising from electrochemical stimuli at the nanoscale.

## 3 Conclusion

In this work, we report on the use of graphitized carbon thin films (anC) as substrates for correlative single‐entity electrochemical, morphological, and spectroscopic characterization. A range of characterization techniques such as electron microscopy (SEM), X‐ray microscopy (STXM, EDS), optical microscopy, scanning probe method (AFM, SECCM) were implemented in a single substrate‐sample, enabling their correlation. Carbon thin film substrates were optimized to meet requirements of nanoelectrochemical measurements, namely low roughness, homogeneous morphology and chemical composition. Notably, these carbon substrates are outstanding for correlative nanoelectrochemistry due to their electrochemical inertness in aqueous electrolytes which enables good contrast for the characterization of a variety of nanostructures. The anC substrates allowed and provided entity/substrate contrast in combination with all the different characterization techniques implemented, while still offering good conductivity for nanoelectrochemical characterization via SECCM. Further, the anC substrate synthesis method enables batch fabrication of substrates, thereby unlocking batch experimental designs for correlative‐SEE.

Encapsulated metal nanoparticles, carbon nanocubes, and 2D materials (MXenes) were characterized as proof‐of‐concept to exemplify the efficacy of these carbon substrates for correlative electrochemical, morphological, and spectroscopic characterization on single entities. Using the chemical sensitivity of SXM, we were able to map the distribution of electrolyte ions over MXene flakes that were probed via SECCM. We anticipate that such carbon thin films will accelerate the use of correlative studies for an in‐depth understanding of the electrochemical response of nanomaterials.

## 4 Experimental Section

### Fabrication of Carbon Thin Film Electrodes

Carbon materials were synthesized via sputtering deposition using Si and SiO_2_/Si wafers as substrates using previously described protocols.^[^ ^13^ ^]^ Briefly, substrates were first cleaned with piranha solution (3:1 H_2_SO_4_/H_2_O_2_ *CAUTION: piranha solution is a strong oxidant which may react explosively with organics*), then rinsed and dried under a stream of nitrogen prior to sputter deposition. Sputtering was carried out in a dc‐magnetron sputtering chamber with a base pressure <2 × 10^−6^ mbar using a graphite target (Lesker) and either pure Ar or 2% N_2_/Ar (by flow) at 50 sccm, to deposit nitrogen‐free or nitrogenated amorphous carbon, respectively. Carbon thin‐film electrodes were obtained after annealing the above sputtered films at 900 °C: anC and anC:N_P_ were obtained from deposition in Ar followed by annealing under N_2_ flow (200 sccm, 900 °C, 60 min), and under NH_3_/N_2_ flow (100 sccm ea., 30 min) followed by N_2_ (200 sccm, 10 min), respectively. anC:N_G_ was obtained from deposition in 2% N_2_/Ar after annealing under N_2_ flow (200 sccm, 900 °C, 60 min). All samples were cooled to room temperature under N_2_ flow prior to air exposure.

### Nanomaterials Preparation

Ni nanoparticles synthesized via chemical reduction in the absence of any ligands/capping shell were obtained commercially (PlasmaChem GmbH). Nanoparticles were used without further purification to prepare 0.04 mg mL^−1^ drop casting dispersions in deionized water via serial dilutions.

Carbon nanocubes were synthesized by chemical vapor deposition using triethylenetriamine (TETA) (Fluka, Honeywell International Inc., Charlotte, North Carolina, USA) as precursor, ferrocene (Sigma‐Aldrich) as catalyst and MgO as substrate. MgO was prepared by a sol‐gel method from oxalic acid (Sigma‐Aldrich) and magnesium acetate (AnalaR, HopkinWilliams Ltd., UK) (1:1 oxalic acid: magnesium acetate by mol.), followed by calcination 4 h at 800 °C (N_2_, 100 cm^3^ min^−1^, ramp 11 °C min^−1^). The MgO (0.3 g) was inserted into a quartz reactor and heated up 800 °C (N_2_, 95 cm^3^ min^−1^, ramp 5 °C min^−1^). When this temperature was reached, a solution of 2.5% wt. ferrocene in TETA was added dropwise (flux = 2.6 cm^3^ h^−1^) over 70 min. At the end of the dripping, the reactor was quenched in air. The amorphous part of the carbon was eliminated by calcination at 400 °C in static air (2 h). To remove MgO the product was lixiviated by sonication in 0.5 m H_2_SO_4_ (Sigma‐Aldrich) at room temperature (90 min). Finally, after washing with water and filtering (Durapore filters, 0.45 µm, Millipore), nanocubes were dried overnight at 110 °C under nitrogen flux. The nanocubes were then used to prepare 0.12 mg mL^−1^ drop casting dispersions via serial dilutions in deionized water.

Few‐layered HF‐etched Ti_3_C_2_T*_x_* MXene were used for SECCM‐STXM experiments. The synthesis of multilayered Ti_3_C_2_T*_x_* MXene powders, used for HF‐etched Ti_3_C_2_T*_x_* MXene, followed the procedure proposed by Mathis et al.^[^ ^30^ ^]^ MXene delamination, as reported in prior work,^[^ ^31^ ^]^ forms a colloidal solution; this solution diluted in water was drop‐cast on SiN windows (Norcada) coated with the thin‐film carbon electrode substrate.

### Characterization

AFM characterization was carried out on a Park NX10 instrument (Park Systems, South Korea). Images were obtained in a non‐contact mode (NCM) with a PPP‐NCHR cantilever type (force constant = 42 N/m, resonance frequency = 330 kHz, Nanosensors). SEM images were acquired with a ZEISS Ultra Plus field‐emision SEM with the secondary electron detectors, SE2 and In‐Lens. Energy dispersive X‐ray spectroscopy (EDX) was performed on the same Zeiss Ultra Plus field‐emission SEM with a 20 mm^2^ Oxford Inca EDX detector. High Resolution Transmission Electron Microscope (HR‐TEM) was carried out on a HR‐TEM FEI Technai F20 Field Emission Gun (FEG) microscope (acceleration potential: 200 kV). Samples were dispersed in ethanol and drop cast on Cu grids covered with Formvar (300 mesh, Electron Microscopy Sciences). Raman characterization was carried out using a Witec alpha 300R confocal scanning Raman system with an excitation wavelength of 532 nm; Raman imaging was carried out over 100 × 100 µm^2^ regions with 33 × 33 pixel resolution. Analysis of peak areas was performed using Witec Project 5.1 software.

SXM measurements were performed at the ultra‐high vacuum scanning X‐ray microscopy (UHV‐SXM) “MAXYMUS” microscope endstation at the UE46‐PGM2 undulator beamline at HZB/BESSY II. Surface‐sensitive total electron yield (TEY) measurements were conducted simultaneously with standard transmission measurements by amplifying the sample current using a commercial amplifier from FEMTO Messtechnik GmbH, then converting it to corresponding frequency values.

### SECCM Electrochemistry Studies

Single‐barrelled nanopipettes were used as SECCM probes. These were fabricated from single‐barrelled borosilicate capillaries (1.5 mm O.D and 0.86 mm I.D., BF150‐86‐7.5, Sutter Instrument, USA) using a laser puller (P‐2000, Sutter Instrument, USA). A pipette filler (MicroFil MF34G‐5, World Precision Instruments, USA) was used to introduce the electrolyte followed by insertion of the quasi reference counter electrode (QRCE). Two types of QRCE were used for the experiments, as noted in each case: a leak‐free Ag/AgCl electrode (LF‐1‐45, Innovative Instruments, Inc., USA) and a Pd‐H electrode. The Pd‐H was fabricated from a Pd wire (0.25 mm OD, PD005130, Goodfellow, UK) by biasing it at −3 V vs. a Pt counter electrode for 15 min in a solution of a strong acid (H_2_SO_4_ or HClO_4_).^[^ ^2f^ ^]^

SECCM measurements were conducted using two types of instruments. SECCM on Ni@C nanoparticles was performed using a Park NX10 (Park Systems, South Korea). SECCM of nanocubes and MXenes was conducted in a custom‐built system as previously described,^[^ ^32^ ^]^ equipped with a commercial transimpedance amplifier (DDPCA‐300, FEMTO, Germany). In both instruments and for all experiments, SECCM hopping mode was employed to map the electrochemical response across a predefined grid of points. Hopping mode involved vertically approaching the pipette to the sample surface at a speed of 0.3 µm s^−1^, until contact was established between the nanopipette droplet and the sample surface. Contact detection was achieved by monitoring a current threshold, which was exceeded upon the appearance of a double‐layer charging current due to droplet cell formation (threshold set at ±3 pA for Park NX10 equipment and ±10 pA for the custom‐built SECCM). Following pipette approach, voltammetry cycles were recorded, and subsequently, the pipette was retracted and repositioned to the next sample point on the predefined grid.

### Statistical Analysis

Analysis of nanoparticle frequencies are detailed in Sections S3 and S4 (Supporting Information).

## Conflict of Interest

Kim McKelvey reports that equipment used for some of the reported experiments was provided by Park Systems Corp.

## Supporting information

Supporting Information

## Data Availability

The data that support the findings of this study are available from the corresponding author upon reasonable request.
