# Supplementary material for: Carbon Thin‐Film Electrodes as High‐Performing Substrates for Correlative Single Entity Electrochemistry
Source: Small Methods. 2024 Aug 19;9(1):2400639. doi: 10.1002/smtd.202400639 (PMC11740950; doi:10.1002/smtd.202400639)
Supplement: Supplementary file 1 — Supporting Information [file SMTD-9-2400639-s001.pdf]

small methods

Supporting Information

for *Small Methods*, DOI 10.1002/smtd.202400639

Carbon Thin-Film Electrodes as High-Performing Substrates for Correlative Single Entity Electrochemistry

Marc Brunet Cabré, Christian Schröder, Filippo Pota, Maida A. Costa de Oliveira, Hugo Nolan, Lua Henderson, Laurence Brazel, Dahnan Spurling, Valeria Nicolosi, Pietro Martinuz, Mariangela Longhi, Faidra Amargianou, Peer Bärmann, Tristan Petit, Kim McKelvey* and Paula E. Colavita**

Supporting Information for:

Carbon Thin-Film Electrodes as High-Performing Substrates for Correlative Single-Entity Electrochemistry

Marc Brunet Cabré, Christian Schröder, Filippo Pota, Maida A. Costa de Oliveira, Hugo Nolan, Lua Henderson, Laurence Brazel, Dahnan Spurling, Valeria Nicolosi, Pietro Martinuz, Mariangela Longhi, Faidra Amargianou, Peer Bärmann, Tristan Petit, Kim McKelvey,* Paula E. Colavita **

Marc Brunet Cabré, Christian Schröder, Filippo Pota, Maida A. Costa de Oliveira, Hugo Nolan, Lua Henderson, Laurence Brazel, Kim McKelvey, Paula E. Colavita
School of Chemistry, Trinity College Dublin, Dublin 2, Ireland.
Email: colavitp@tcd.ie

Pietro Martinuz, Mariangela Longhi
Università degli Studi di Milano, Dipartimento di Chimica, Via Golgi 19, 20133 Milano, Italy.

Faidra Amargianou, Peer Bärmann, Tristan Petit
Helmholtz-Zentrum Berlin für Materialienund Energie GmbH (HZB), Albert-Einstein-Straße15, 12489 Berlin (Germany).
Email: tristan.petit@helmholtz-berlin.de

Kim McKelvey
MacDiarmid Institute for Advanced Materials and Nanotechnology, School of Chemical and Physical Sciences, Victoria University of Wellington, Wellington 6012, New Zealand
Email: kim.mckelvey@vuw.ac.nz

Dahnan Spurling, Valeria Nicolosi
CRANN and AMBER Research Centres, Trinity College Dublin, Dublin 2, Ireland.

Supporting Information Contents

SI-1: Carbon substrate morphology characterization 4
Figure S1: AFM images of carbon roughness. 4
Figure S2: AFM images of step edge and profiles. 4
Figure S3: SEM images of film cross sections. 5
Figure S4: Raman maps of carbon substrates. 5
SI-2: Electrochemical characterization of bare substrates 6
Figure S5: Additional voltammograms using diagnostic redox probes. 6
Figure S6: Average voltammograms and current distributions. 7
Figure S7: Additional voltammograms in supporting electrolyte. 7
SI-3: Ni@C NPs morphology and particle distribution analysis 8
Figure S8: AFM images of Ni@C nanostructures. 8
Figure S9: Ni NP distributions. 9
Figure S10: AFM image processing to determine NP occupation. 9
Table S1: Calculated probability of contacting NPs. 10
SI-4: Ni@C NPs SECCM electrochemical data analysis 11
Figure S11: SECCM scheme, voltammograms and current maps. 11
Figure S12: SEM image of probed grid. 12
Figure S13: SEM-EDX images of Ni@C samples. 13
Figure S14: SEM images of grid and surrounding areas. 14
Table S2: Summary of probabilities of contacting NPs. 15
SI-5: Morphology of isolated MXene ($Ti_3C_2T_x$) on anC by AFM 16
Figure S15: AFM image of MXene flakes 16
SI-6: SECCM electrochemical data analysis of MXene ($Ti_3C_2T_x$) 16
Figure S16: Optical image of MXene flakes and grid. 16
Figure S17: Voltammograms at each SECCM grid point. 17
Figure S18: Optical and SXM images of MXene flakes and grid. 18
Figure S19: Transmission, TEY and SXM spectra of MXene flakes. 19

SI-1: Carbon substrate morphology characterization

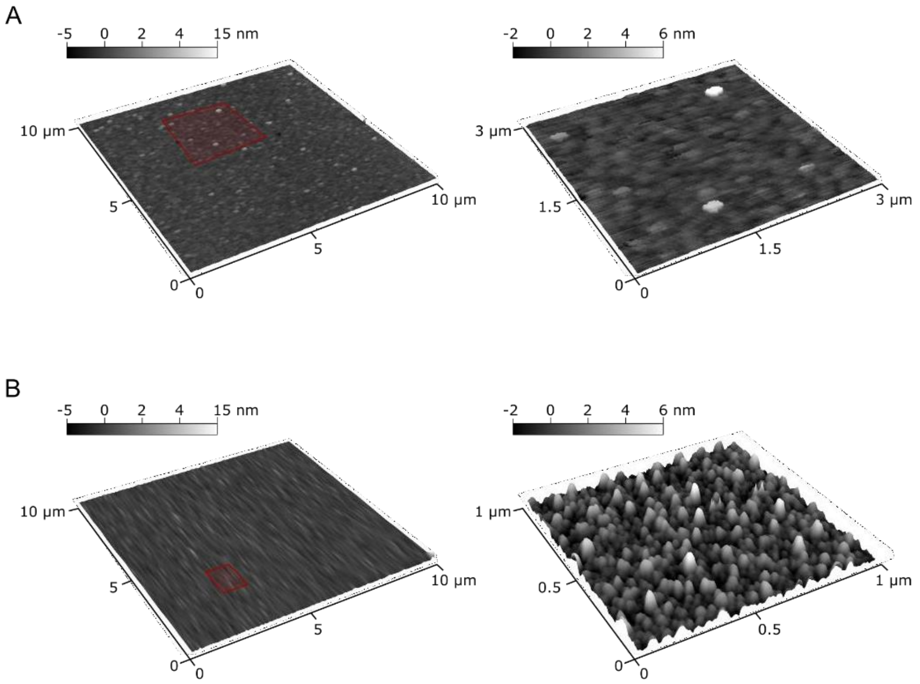

Figure S1: AFM characterization of A) anC and B) anC:Np surface. On the left, area of 100 µm2 scanned with a red square indicating the area were greater magnification image, right, is obtained. All AFM images are plotted with Z-scale magnified ×10 respect X and Y.

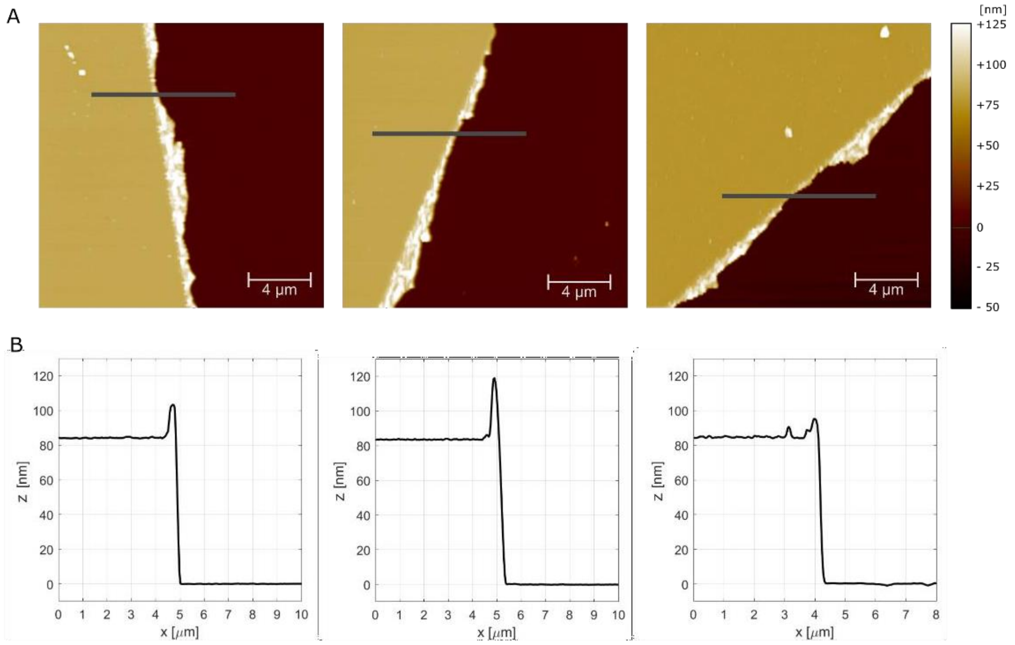

Figure S2: AFM characterization of anC thickness by evaluating the step profile from anC to supporting Si wafer substrate. **(A)** AFM images of three step regions located separated at >1mm distance. **(B)** Step line profiles as indicated in grey line in (A).

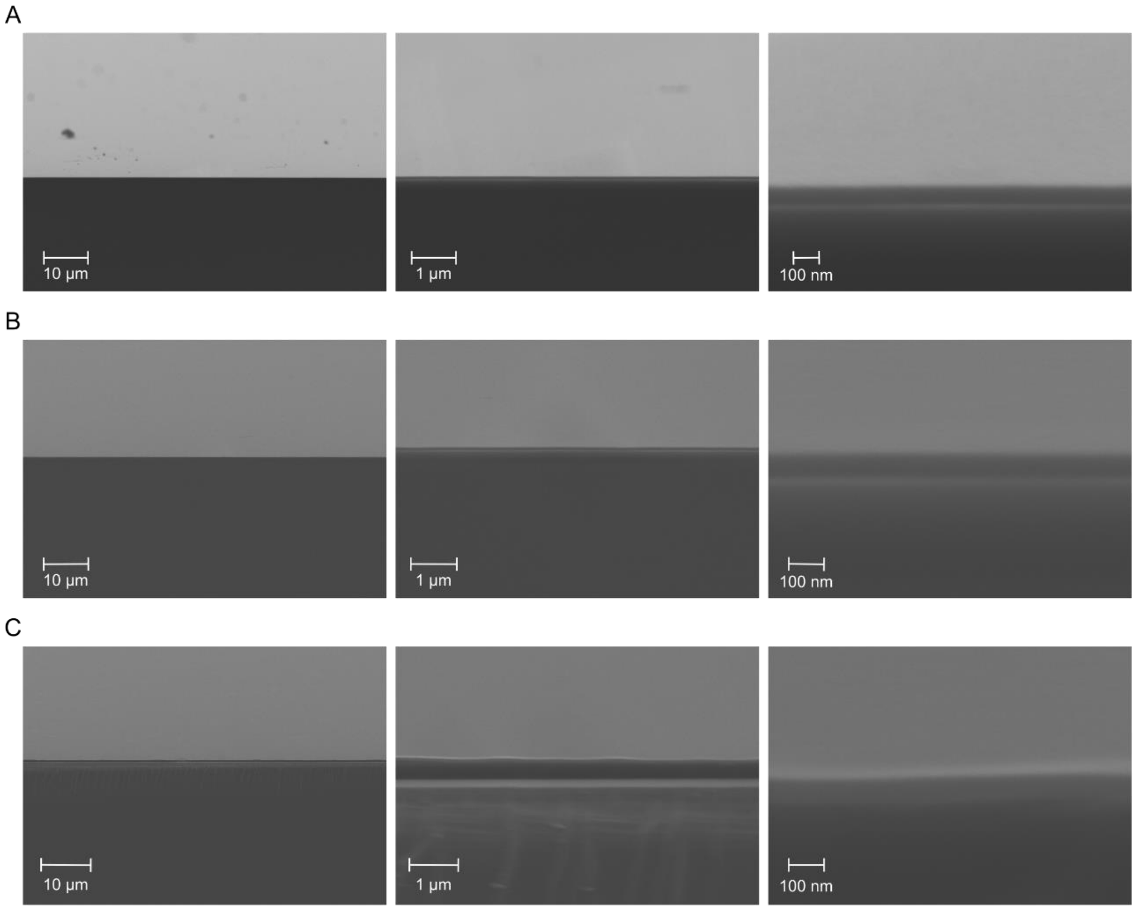

Figure S3: SEM images of cross sections of **(A)** anC, **(B)** anC:N_G and **(C)** anC:N_P at three different magnifications.

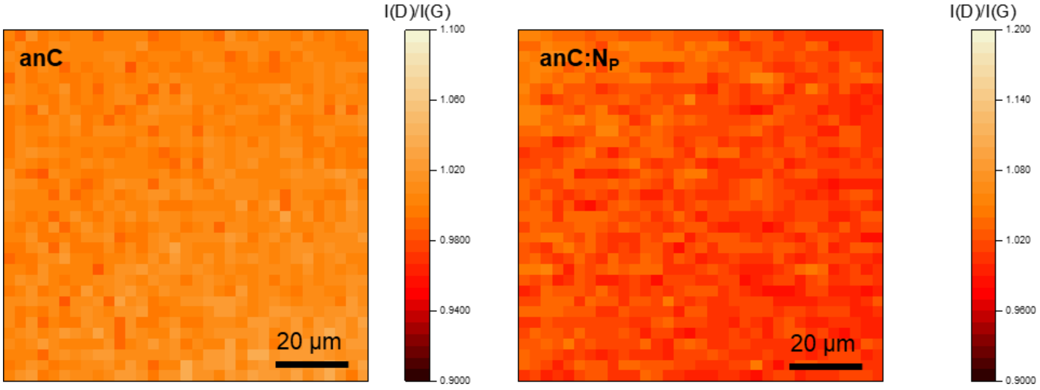

Figure S4: Raman mapping of I(D)/I(G) ratio across anC and anC:N_P carbon thin film surfaces.

SI-2: Electrochemical characterization of bare substrates

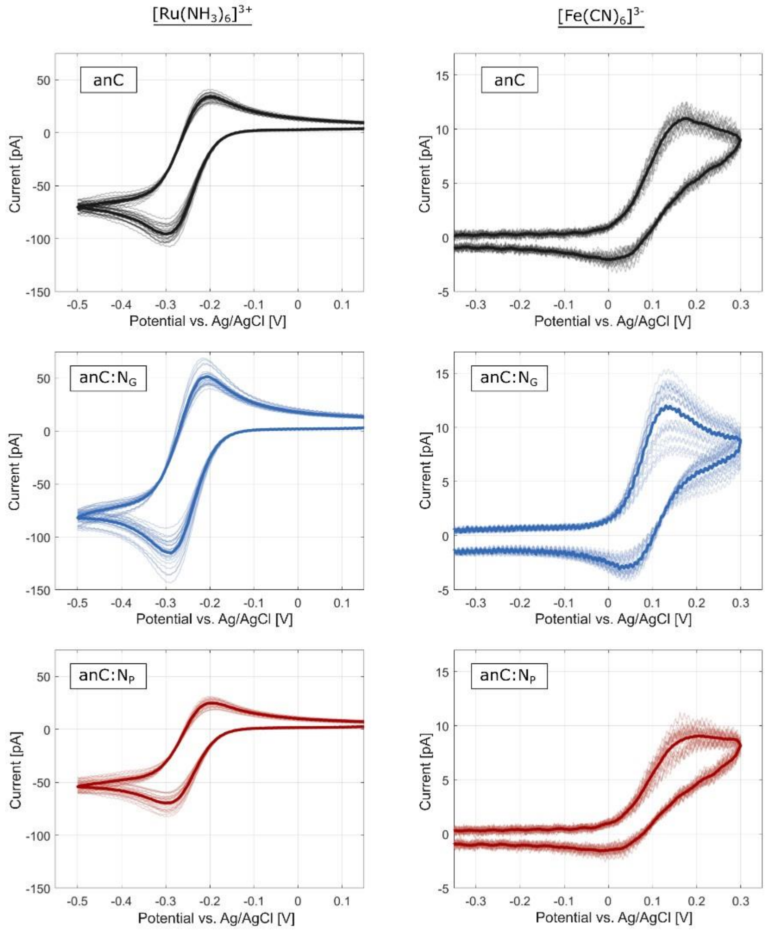

Figure S5: Voltammograms of $Ru(NH_3)_6^{3+/2+}$, and $Fe(CN)_6^{3-/4-}$, redox probes on bare anC, anC:N_G and anC:N_P. Thin lines correspond to the CV for each of the 25 points probed in a grid; thick lines represent the mean response calculated for each substrate. All voltammograms were collected at 0.500 V/s in 10 mM KCl containing 4.0 mM $Ru(NH_3)_6^{3+}$ and 1.0 mM $Fe(CN)_6^{3-}$.

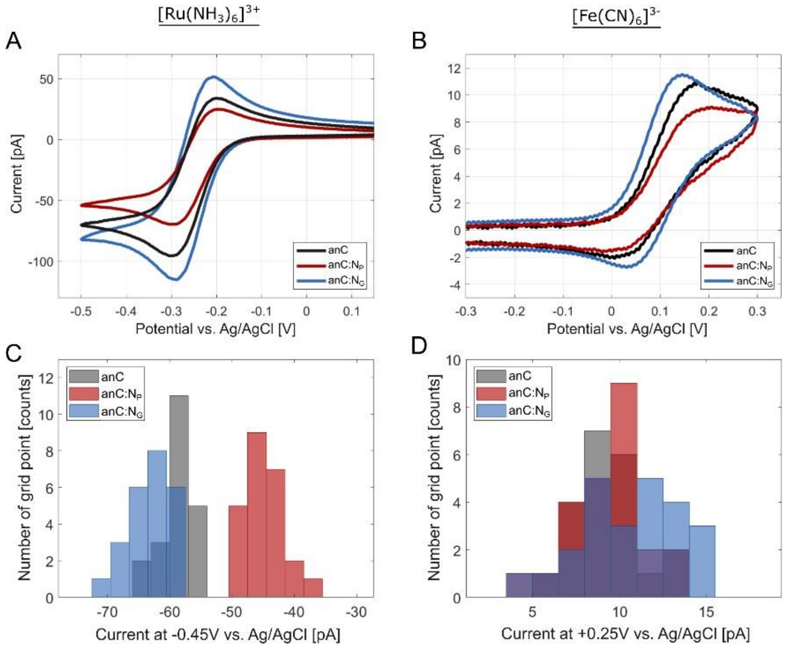

Figure S6: Averaged voltammograms over the 25 SECCM grid points for **(A)** outer-sphere, $[Ru(NH_3)_6]^{2+/3+}$, and **(B)** inner-sphere, $[Fe(CN)_6]^{3/4-}$, redox probes on bare anC, anC:N$_G$ and anC:N$_P$. **(C)** and **(D)** Histogram of diffusion limiting currents (current at -0.45 V vs. Ag/AgCl) for the $[Ru(NH_3)_6]^{2+/3+}$ redox couple.

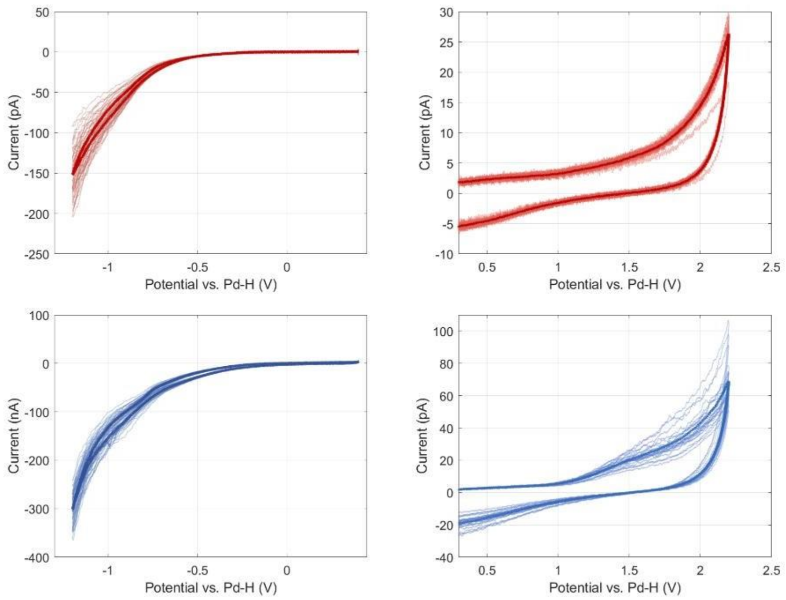

Figure S7: CVs obtained via SECCM in 20 mM H_2SO_4 supporting electrolyte at 0.5 V s^{-1} at the cathodic and anodic limits of the potential window using bare anC:N$_P$ (red) and anC:N$_G$ (blue) substrates as working electrodes. Thin lines correspond to CVs at each of the 25 grid points probed; thick lines represent the calculated mean response for the substrate.

SI-3: Ni@C NPs morphology and particle distribution analysis

The height distribution of Ni@C nanostructures was obtained by analyzing particle heights over four different AFM images (Figure S7), each collected on different sample regions and each with an area of 100 μm^2.

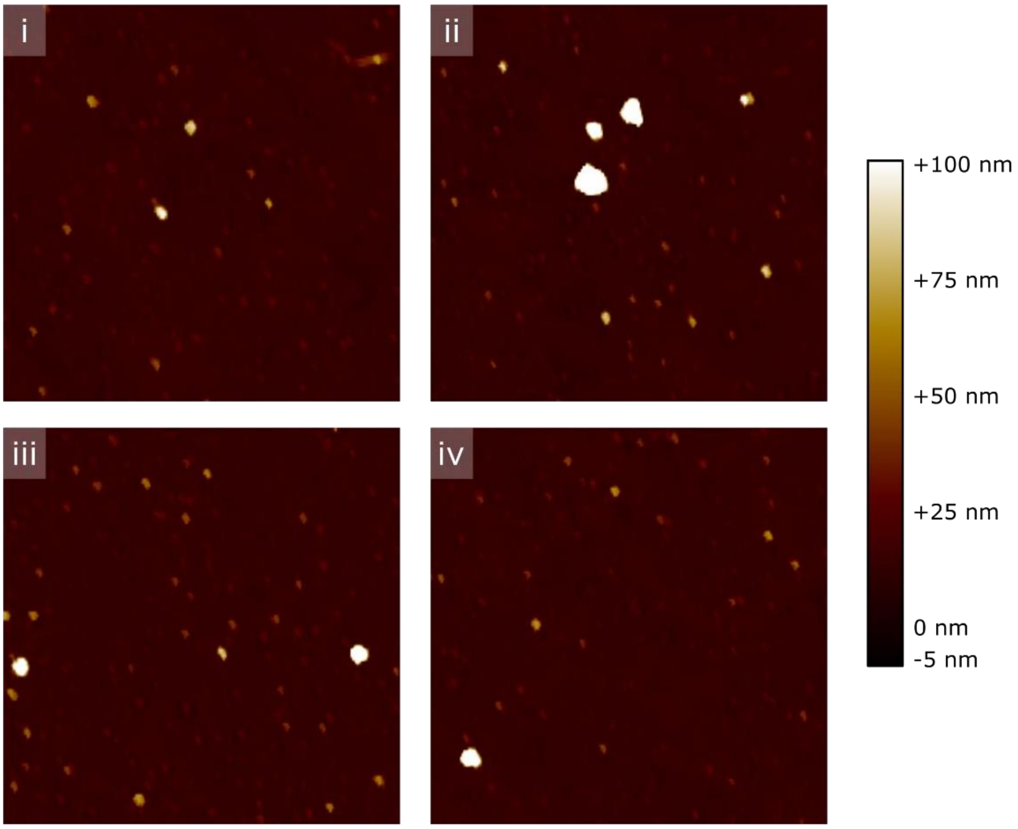

Figure S8: AFM images of four different regions (i, ii, iii and iv) containing Ni@C NPs. All images are 10 × 10 μm^2 in size.

Particle analysis was performed by identifying domains that exceed a given peak-to-valley height threshold. Figure S8 shows the height distributions obtained with height thresholds of 5 and 20 nm. For a 5 nm threshold, a large population peak between 5 and 20 nm is observed, followed by another peak at 20-40 nm. Smaller populations are observed at ca. 60 and 90 nm. The nominal average size of the commercially sourced Ni NPs used in our experiments is 50 nm but the final size of encapsulated Ni@C structures is affected by the a-C coating (10 nm thickness). This suggests that the population 20-40 nm in peak-to-valley height in Figure S7 is therefore representative of the Ni@C nanostructures, while the population at <20 nm corresponds to morphological features arising from sample handling/preparation outside of a cleanroom environement (e.g. dropcasting, particulates). Populations with larger heights (60, 90 and > 120 nm), on the other hand, are likely to originate from agglomerates of more than

one Ni@C NP. Therefore, to exclude features <20 nm in size we selected a peak-to-valley threshold of 20 nm to obtain size distributions representative of Ni@C nanostructures.

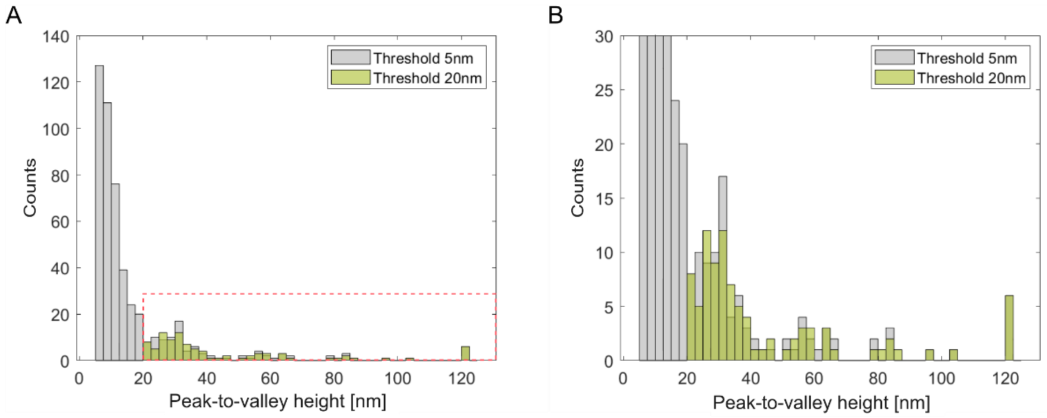

Figure S9: (A) Population distributions and **(B)** their expanded version obtained with peak-to-valley height thresholds of 5 nm (gray) and 20 nm (green).

The probability of finding one or more Ni@C NPs over 1 μm^2 was estimated from AFM images using two approaches. First, a method of particle counting over quadrants was adopted. The AFM images shown in Figure S8 were converted into binary images using a masking threshold of 20 nm in height (criterion for detection of Ni@C NPs, as above). Images were then divided into 1 μm^2 quadrants and the number of quadrants with active pixels was divided over the total number of quadrants (100 per image), yielding probability estimates as in Table S1. An example of this process is shown in Figure S9 for one of the images used.

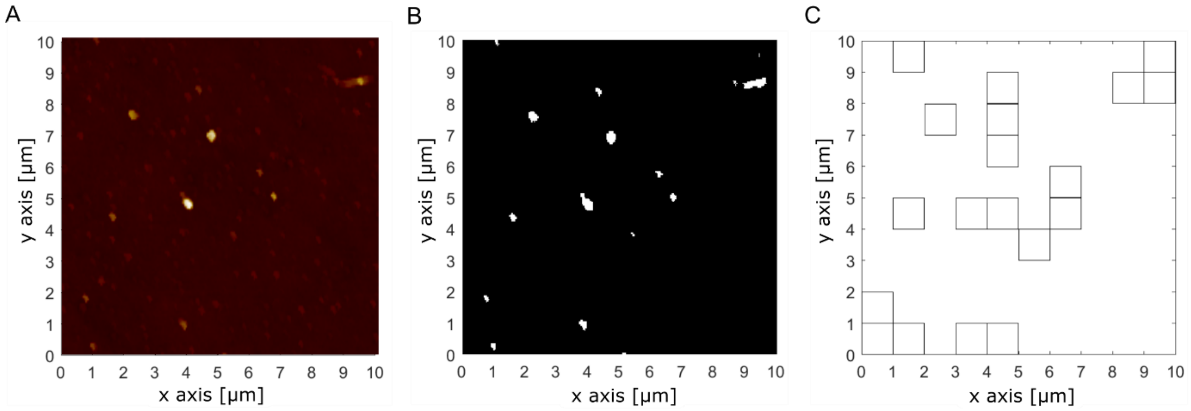

Figure S10: **(A)** AFM height image and **(B)** the corresponding binary image generated by applying a 20 nm threshold. **(C)** Quadrants of 1 μm^2 are containing Ni@C NPs.

Second, the probability was calculated using a particle counting method based on random sampling of the images. The contact droplet area was simulated as a circular mask - in analogy to the circular shape of an SECCM droplet cell - that could be positioned at random coordinates across the image domain. The following steps were used:

1. A mask consisting of a 1 μm^2 circular area was defined with its center on the AFM image at *x,y* coordinates generated from a random uniform distribution, as follows:

$$Area\ droplet = 1\ \mu m^2$$

$$r = \sqrt{\frac{Area\ droplet}{\pi}}$$

$$x_{center} \in \mathbb{R}\{r, 10 - r\} \qquad x = \mathbb{R}\{0{,}10\}$$

$$y_{center} \in \mathbb{R}\{r, 10 - r\} \qquad y = \mathbb{R}\{0{,}10\}$$

$$droplet\ cell\ mask \in \mathbb{R}\{(x - x_{center})^2 + (y - y_{center})^2 \leq r^2\}$$

2. The domain of points resulting from an overlap between the binary image and the droplet mask was analyzed and a particle was counted if 3 or more pixels were active within the domain.
3. Processes 1 and 2 were repeated 20000 times and the probability of contacting a Ni@C NPs within the AFM image was then calculated as:

$$prob.contact = \frac{times\ C@NiNPs\ contacted}{total\ attemps}$$

Table S1 summarizes results generated with these two approaches and indicates that very similar estimates are obtained independently of the approach adopted. However, the estimate was found to be strongly dependent on the AFM image analyzed, thus suggesting that drop-casting yields a non-uniform particle density, as discussed in the main text.

Table S1: Probability of concting Ni@C NPs decteded on the AFM images (displayed in Figure S6) by quadrant and randomly distributed SECCM droplet cell methods.

AFM image	Probability of Ni@C NPs per quadrant	Probability of randomly contacting Ni@C NPs with a circular 1 μm^2 SECCM cell
i	0.19	0.135
ii	0.39	0.358
iii	0.40	0.357
iv	0.27	0.279
**mean ± std**	**0.30 ± 0.10**	**0.28 ± 0.10**

SI-4: Ni@C NPs SECCM electrochemical data analysis

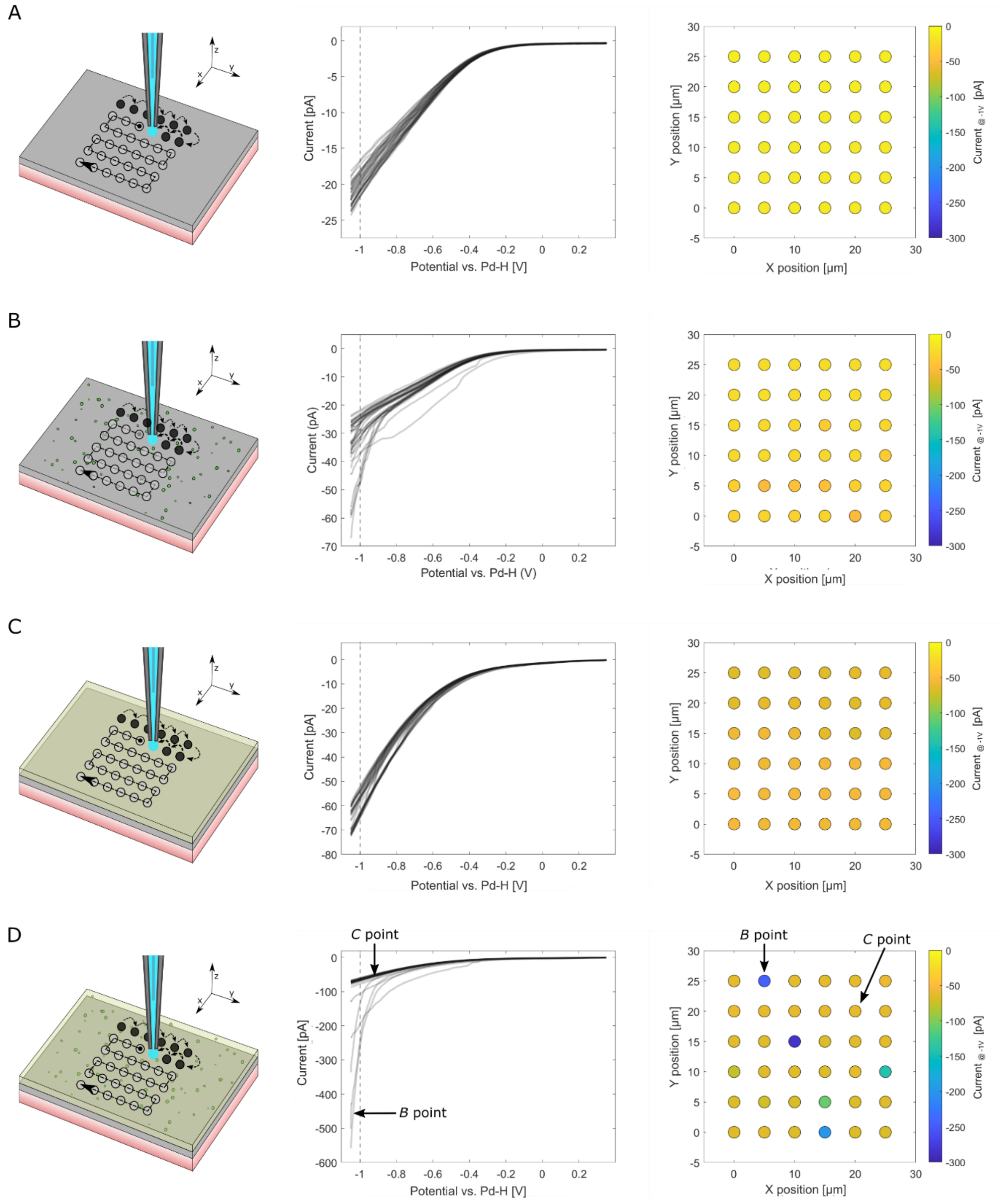

Figure S11: On the left, scheme the measurement of a 6×6 SECCM grid, on which a voltammogram is recorded for each point probed; On the centre, overlapped plot of the forward cathodic scans of each the 36 points probed; On the right, electrochemical mapping showing in coloured scalebar the current at -1 V; for the samples **A)** bare anC, **B)** bare Ni NPs on anC, **C)** a-C onto anC and **D)** Ni@C NPs on anC.

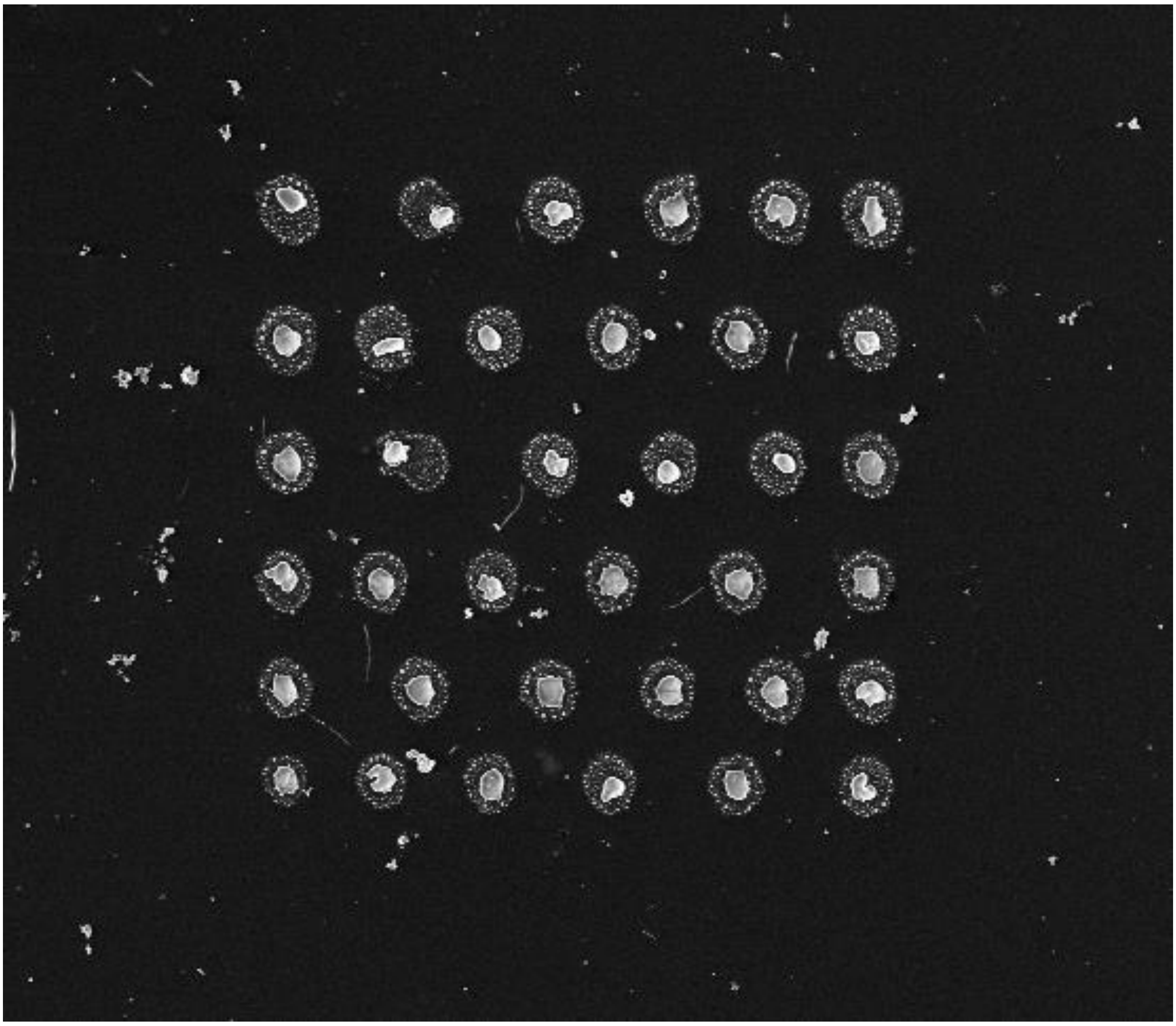

Figure S12: SEM image of the region of Ni@C NPs sample where SECCM grid was conducted, showing the encapsulated Ni NPs entities over the anC substrate and the SECCM droplet cell residues. SEM image has not been coloured or modify from acquisition. Collected in InLense detector (major contribution of backscattered electrons) with 3 kV beam. Image size of 68x60 µm.

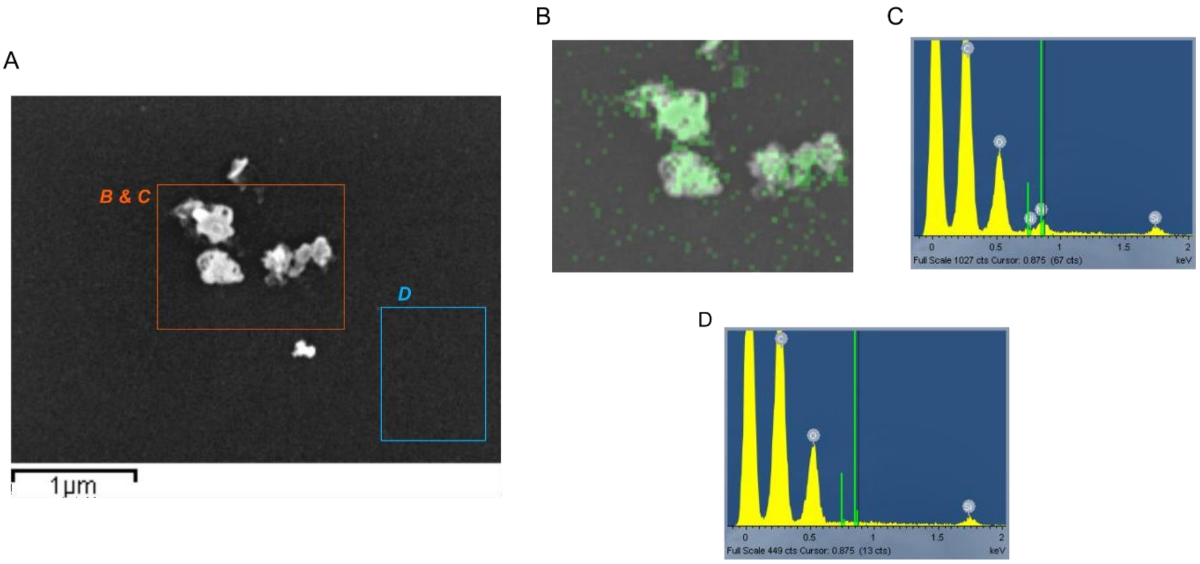

Figure S13: (A) SEM image of entities over anC substrates assigned to encapsulated Ni@C. SEM image outside of the sample region probed with SECCM. Orange and blue squares indicate regions where EDS spectra and mapping were performed. **(B)** EDS coloured mapping of the Ni concentration overlapped with the corresponding SEM image, showing that Ni-rich features match morphological freatures assigned to Ni@C. **(C)** EDS spectrum showing the presence of Ni peaks in region A (orange frame). **(D)** EDS spectrum showing no Ni peaks in region D (blue frame).

The probability of contacting a Ni@C NPs on region measured by SECCM was evaluated via analysis of the SEM images using the random sampling method described in Section SI-3 and a circular mask 8.5 µm^2 in size. The AFM the criterion for implementing a binary image function was based on height threshold, however given that SEM images do not provide such morphological information, a brightness contrast threshold was used instead. Given that the backscatterred electron image contrast is related to atomic number, this can be expected to be a good criterion for the identification of Ni NP core structures. The binary SEM image was calculated using Otsu's method;[1] the orginal SEM image and the binary images used for probability determination are shown in Figure S14. Notice that 4 binary images were generated from 4 regions of the SEM image surrounding the SECCM grid area. An example of the mask located at random x_{center},y_{center} coordinates overlaped with binary SEM image is displayed in Figure S14E, while probability estimates for each image are reported in Table S2.

[1] Otsu, N. A Threshold Selection Method from Gray-Level Histograms. IEEE Transactions on Systems, Man, and Cybernetics 1979, 9 (1), 62-66. DOI: 10.1109/TSMC.1979.4310076.

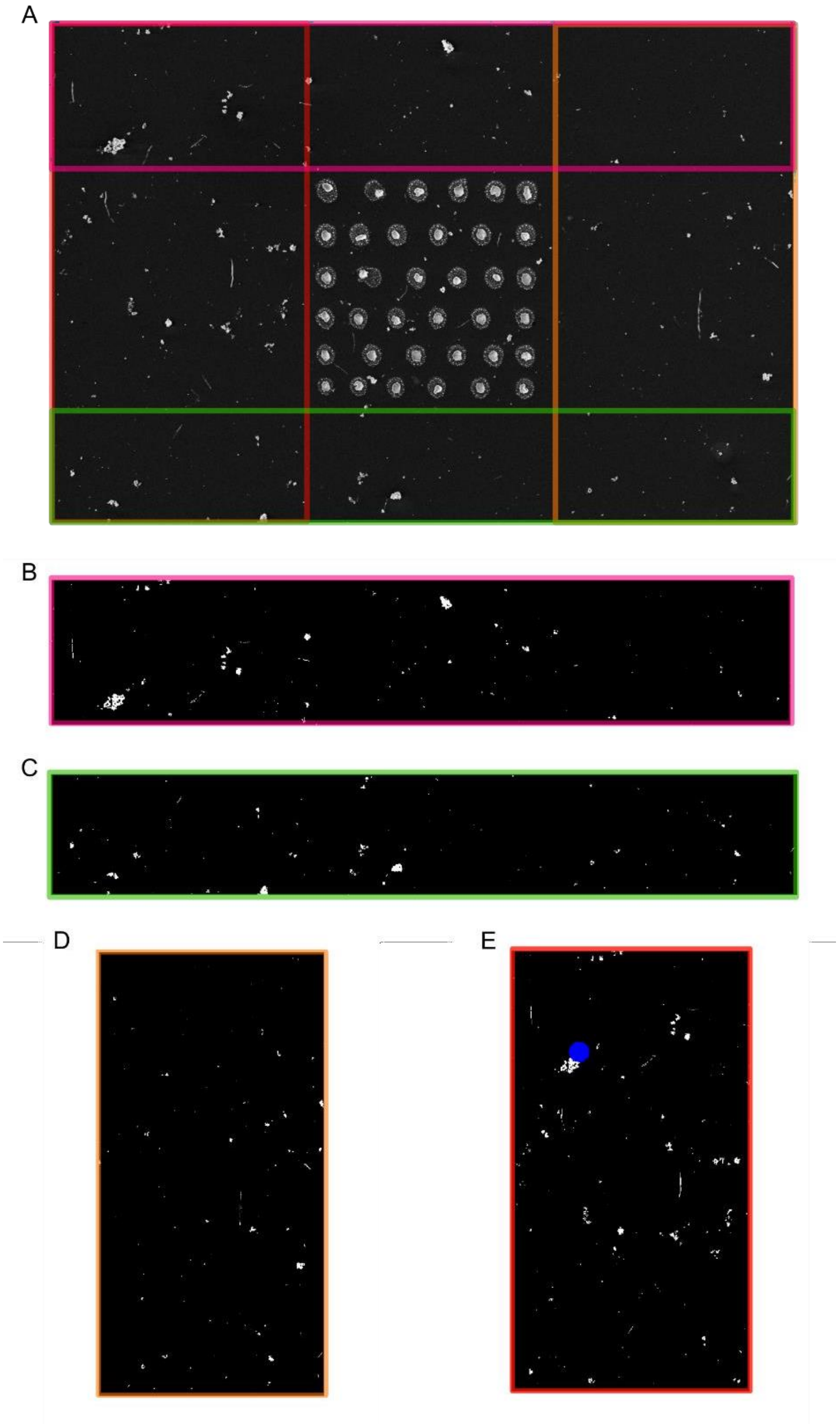

Figure S14: (A) SEM image indicating areas around the SECCM grid (red, pink, orange and green rectangle) that were selected for probability calculations. **(B)**, **(C)**, **(D)** and **(E)** are the binary images obtained by applying a 0.5 brigthness threshold to the areas identified in (A). An SEM binary image showing an overlap with a simulated SECCM droplet cell mask with a 8.5 µm^2 area is shown in panel (E) (blue circle).

Table S2: Probability of contacting Ni@C NPs from the subregions identified in the SEM images, as displayed in Figure S14.

**Subregion Image (color code)**	**Probability of randomly contacting Ni@C NPs with a 8.5 μm^2 SECCM droplet cell**
Red	0.221
Pink	0.157
Orange	0.128
Green	0.163
**mean ± std**	**0.167 ± 0.039**

SI-5: Morphology of isolated MXene ($Ti_3C_2T_x$) on anC by AFM

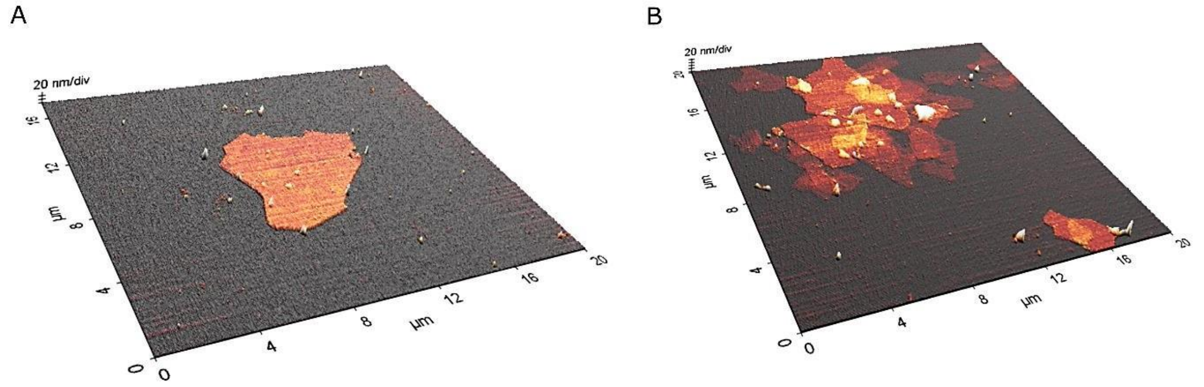

Figure S15: AFM images of two different regions of anC substrate with dropcasted MXene flakes. **(A)** Displays an isolated flake on anC and (**B)** displays multiple flakes which present partial stacking between them.

SI-6: SECCM electrochemical data analysis of MXene ($Ti_3C_2T_x$)

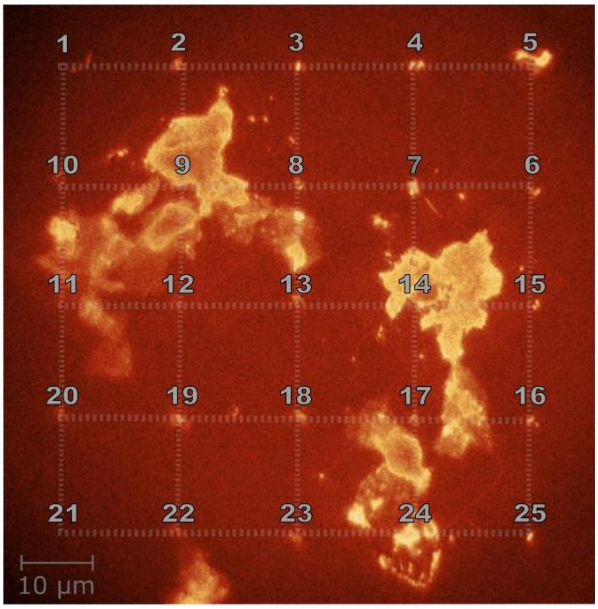

Figure S16: Optical image of MXenes drop-cast on carbon substrates in the region measured by SECCM, showing the grid points (dashed lines included as visual guide) with MXene flakes (lighter colour) over anC/SiN substrate. Each of the points probed by SECCM is labelled with a number. The corresponding voltammograms are displayed in Figure S17.

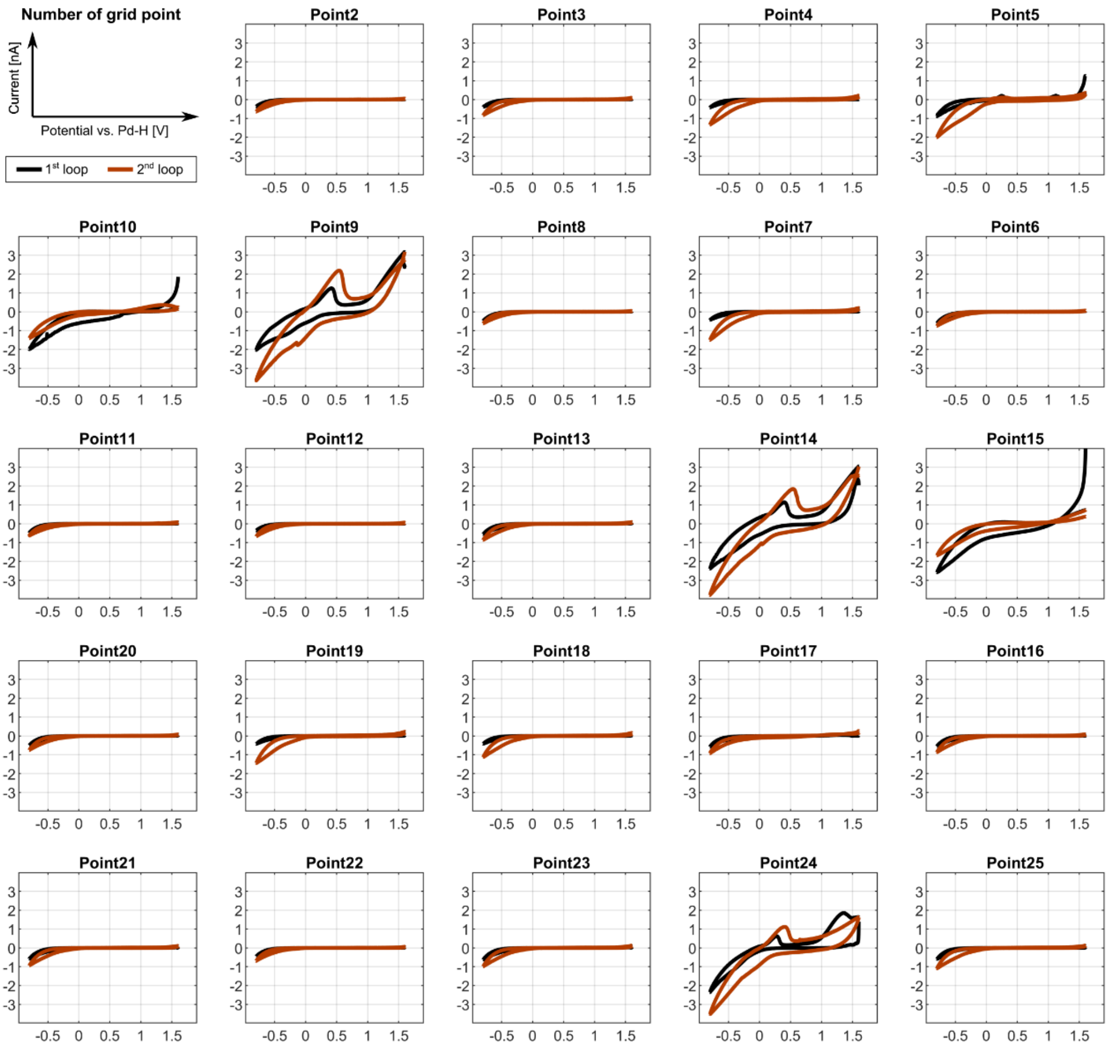

Figure S17: Voltammograms obtained for each of the points probed in the SECCM grid, showing distinct behaviour depending on whether they contact the anC substrate and/or MXene flakes, as shown in Figure S16. On each point two cycles were recorded, starting from anodic potentials. The first cycle is displayed in black and the second in orange. No voltammogram was recorded at the first point, hence the empty space shows only the figure legend.

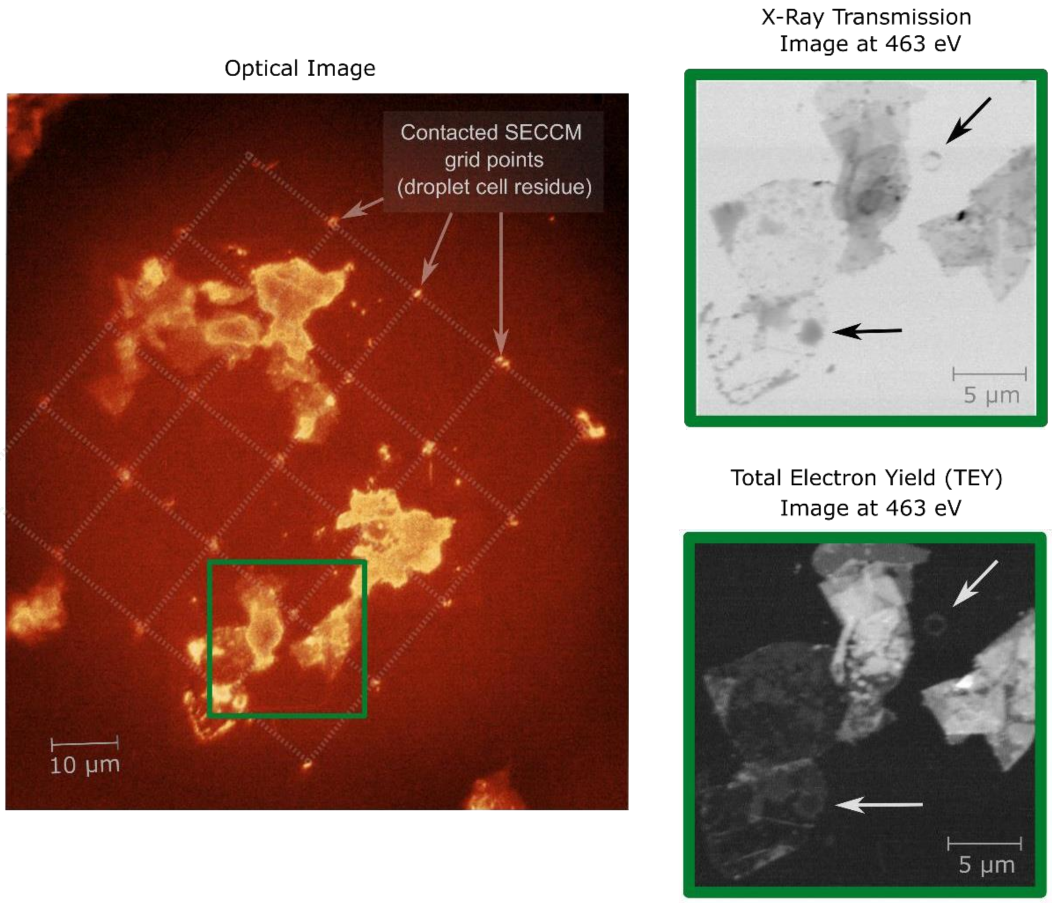

Figure S18: Left: optical image of a region probed via SECCM showing the probed grid points (dashed lines between points have been added as a visual guide), the MXene flakes (lighter colour) and the anC/SiN substrate (dark red colour). Right: transmission and TEY images of $Ti_3C_2T_x$ MXene at 463 eV obtained over the region framed by a green squares in the optical image. The visible circular features indicated with arrows in transmission and TEY images correspond to droplet cell residues.

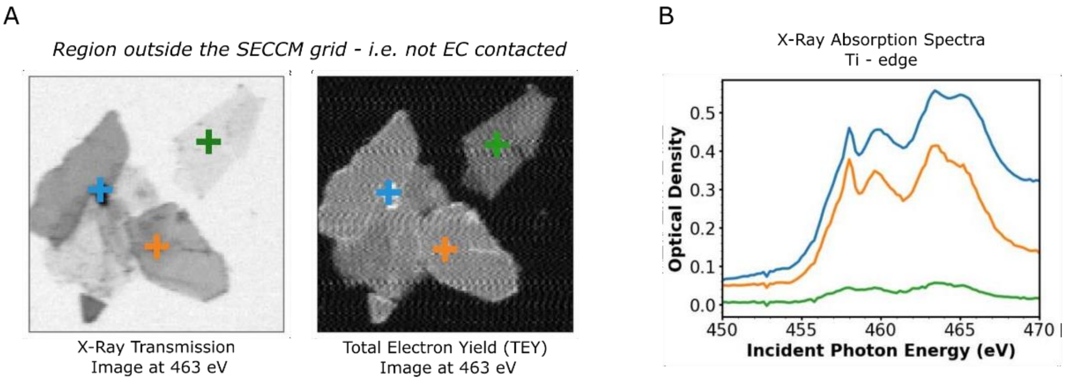

Figure S19: (A) Transmission and TEY images of $Ti_3C_2T_x$ MXene at 463 eV obtained for MXene flakes located at positions that were not probed by SECCM. Transmission spectra at the Ti L-edge were obtained at positions indicated with crosses of matching colour.
(B) Ti L-edge spectra at three different positions across the sample.
